# Cell Type- and Sex-Dependent Transcriptome Profiles of Rat Anterior Pituitary Cells

**DOI:** 10.3389/fendo.2019.00623

**Published:** 2019-09-18

**Authors:** Patrick A. Fletcher, Kosara Smiljanic, Rafael Maso Prévide, James R. Iben, Tianwei Li, Milos B. Rokic, Arthur Sherman, Steven L. Coon, Stanko S. Stojilkovic

**Affiliations:** ^1^Laboratory of Biological Modeling, National Institute of Diabetes, Digestive and Kidney Diseases, National Institutes of Health (NIH), Bethesda, MD, United States; ^2^Section on Cellular Signaling, Eunice Kennedy Shriver National Institute of Child Health and Human Development, National Institutes of Health (NIH), Bethesda, MD, United States; ^3^Molecular Genomics Core, Eunice Kennedy Shriver National Institute of Child Health and Human Development, National Institutes of Health (NIH), Bethesda, MD, United States

**Keywords:** pituitary gland, transcriptome, rat, single-cell RNA sequencing, folliculostellate cells, hormone-producing cells, sexual dimorphism

## Abstract

Understanding the physiology and pathology of an organ composed of a variety of cell populations depends critically on genome-wide information on each cell type. Here, we report single-cell transcriptome profiling of over 6,800 freshly dispersed anterior pituitary cells from postpubertal male and female rats. Six pituitary-specific cell types were identified based on known marker genes and characterized: folliculostellate cells and hormone-producing corticotrophs, gonadotrophs, thyrotrophs, somatotrophs, and lactotrophs. Also identified were endothelial and blood cells from the pituitary capillary network. The expression of numerous developmental and neuroendocrine marker genes in both folliculostellate and hormone-producing cells supports that they have a common origin. For several genes, the validity of transcriptome analysis was confirmed by qRT-PCR and single cell immunocytochemistry. Folliculostellate cells exhibit impressive transcriptome diversity, indicating their major roles in production of endogenous ligands and detoxification enzymes, and organization of extracellular matrix. Transcriptome profiles of hormone-producing cells also indicate contributions toward those functions, while also clearly demonstrating their endocrine function. This survey highlights many novel genetic markers contributing to pituitary cell type identity, sexual dimorphism, and function, and points to relationships between hormone-producing and folliculostellate cells.

## Introduction

The pituitary gland is a complex organ composed of anterior, intermediate and posterior lobes. The embryonic development of anterior and intermediate lobes is initiated by formation of a rudimentary Rathke's pouch, followed by development of a definitive pouch, which is critically dependent on expression of LIM homeobox genes *Lhx3* and *Lhx4* (1, 2). At this developmental stage, *Pitx1* and *Pitx2* are also expressed in Rathke's pouch, but not in the adjacent ventral diencephalon, which forms the infundibular process and posterior pituitary (3, 4). Furthermore, Rathke's pouch is mainly composed of progenitor cells expressing *Sox2* and *Sox3*, which are essential for progenitor proliferation and development of all major anterior pituitary cell types downstream of *Lhx3*/*Lhx4* (5–7). This is followed by expression of *Prop1* and other progenitor genes, detachment from the oral ectodomain and generation of three major hormonal cell lineages (8). The expression of *Tbx19* in a fraction of these cells leads to generation of two cell types producing proopiomelanocortin (POMC): corticotrophs (C)—secreting adrenocorticotropin, and melanotrophs—secreting melanocyte-stimulating hormone (9). The expression of *Nr5a1* is necessary for generation of gonadotrophs (G)—secreting luteinizing hormone and follicle-stimulating hormone (10). Finally, from a fraction of cells expressing *Pou1f1* are derived three cell types: thyrotrophs (T)—secreting thyroid-stimulating hormone, somatotrophs (S)—secreting growth hormone, and lactotrophs (L)—secreting prolactin (11). These cell types, known as endocrine, secretory, or hormone-producing cells (HPC), release hormones predominantly through regulated exocytosis. C, G, T, S, and L are located in the anterior lobe, which also contains folliculostellate cells (FSC) and proliferative cells as well as endothelial cells (EC), pericytes, erythrocytes (E), and leukocytes (Le) from the pituitary sinusoidal capillary network. FSC are chromophobes (12), and *S100b* was generally accepted as their marker gene (13). This glia-like cell type has been suggested to be a heterogeneous group serving various functions, including structural, signaling, and supportive roles to HPC function. It has been suggested that a subset of FSC may serve as pituitary progenitor cells (14, 15) and that FSC and HPC could be derived from the same pool of cells (16).

Different methodologies have been used to characterize these cell types. Early work with mixed pituitary cells led to identification of G protein-coupled receptors specific for HPC: C expressing corticotropin-releasing hormone receptor and arginine vasopressin receptor 1B, G expressing gonadotropin-releasing hormone receptor, T and L expressing thyrotropin-releasing hormone receptor, L expressing dopamine receptor D2 receptor, and S expressing growth hormone-releasing hormone receptor and somatostatin receptor 2 (17). Population studies were used to characterize signaling pathways in these cells (18). Immunohistochemical and immunocytochemical methods helped to quantify the number and distribution of specific cell types within the gland and suggested the existence of multihormonal cells (19). Elegant *in vivo* imaging helped to identify networks of FSC and HPC (20), whereas single-cell imaging and patch clamp experiments showed the cell type-specific electrical and calcium signaling patterns in unstimulated and stimulated cells (17). RNA-sequencing analysis in cultured rat anterior pituitary cells was used to identify upregulated genes and a GnRH controlled gene network in G (21), and single cell RNA-sequencing (scRNA-seq) was used to characterize gene expression in LβT2 gonadotroph cells (22, 23), purified mouse gonadotrophs (24), and dispersed male mouse pituitary cells (25). Despite these advances, the gene expression patterns underlying the identity of anterior pituitary cell types, their functions, and their interrelationships remain incompletely understood.

To gain a transcriptome-wide perspective of rat anterior pituitary cell identities and functions, we performed scRNA-seq on freshly dispersed anterior pituitary cells from postpubertal male and female rats. We identified cell type-specific gene expression in HPC and FSC, examined cellular heterogeneity in these populations, and compared them with pituitary-non-specific cell populations: EC, E, and Le. Comparison of males and females, the latter in the diestrus phase of their estrous cycle, allowed us to identify sex-specific gene expression profiles in pituitary-specific cell types. Our differential expression analysis identified several groups of genes significantly upregulated in FSC, HPC, or both: development/differentiation, neuroendocrine, endogenous ligands, detoxification enzymes, extracellular matrix (ECM) proteins, and cell adhesion molecules. We examined in detail the expression pattern of these genes in pituitary-specific cell types. This work provides a comprehensive transcriptional overview of the anterior pituitary gland that will facilitate future experimental and clinical investigations in this field.

## Materials and Methods

### Chemicals

Penicillin/streptomycin and vitamin solution; phosphate buffered saline (PBS); and HEPES-containing M199 were from Gibco (Grand Island, NY). Bovine serum albumin (BSA), fraction V was from MP Biomedicals (Solon, OH). EDTA was from Corning (Manassas, VA), and calcium chloride was from Quality Biological (Gaithersburg, MD). All other chemicals were from Sigma (St. Louis, MO). Rabbit anti-SOX2, rabbit anti-ALDH1A1 and rabbit anti-GATA2 antibodies were from GeneTex (Irvine, CA). Mouse anti-S100B was from Novus Biologicals, LLC (Centennial, CO). Rabbit anti-GH, guinea pig anti-GH, guinea pig anti-PRL and guinea pig anti-ACTH antibodies were from Dr. A. F. Parlow (National Institute of Diabetes and Digestive and Kidney Diseases, National Hormone and Peptide Program, Torrance, CA). Secondary antibodies Alexa Fluor 488 donkey anti-mouse, Alexa Fluor 488 goat anti-rabbit, Alexa Fluor 568 donkey anti-rabbit, Alexa Fluor 568 goat anti-guinea pig antibodies were from Invitrogen (Carlsbad, CA).

### Animals and Dispersion of Pituitary Cells

Experiments were performed with postpubertal female and male Sprague Dawley rats obtained from Taconic Farms (Germantown, NY). Animals were housed under constant conditions of temperature and humidity, with light on between 6 a.m. and 8 p.m. With female rats, experiments were done using animals in the diestrus stage of their estrous cycle or random cycling animals. After decapitation and removal of the brain, the posterior/intermediate lobes of the pituitary were removed and anterior pituitary glands were collected and used for RNA extraction or cell dispersion, the latter done using a trypsin/EDTA based method, as previously described (26). Briefly, pituitaries were quickly collected from postpubertal rats and kept in 199 Hanks medium. Tissue was washed in ice cold PBS (0.3% BSA; 1.26 mM CaCl_2_), chopped in 0.5 × 0.5 mm pieces and incubated in a shaking a water bath with trypsin for 15 min, 50 rpm. Trypsin solution was removed and replaced by trypsin inhibitor for 5 min. The latter was removed and a 2 mM EDTA solution was added for 5 min and then replaced by a 1 mM EDTA solution for 10 min. After these steps tissue was mechanically dispersed, centrifuged, and cells were counted and used for scRNA-seq, qRT-PCR analysis, or immunocytochemistry. All experiments were approved by the NICHD Animal Care and Use Committee (16-041).

### qRT-PCR Analysis

Total RNA was extracted from individual anterior pituitary glands and anterior pituitary cells 30 min after cell dispersion using an RNeasy Plus Mini Kit (Qiagen, Valencia, CA). RNA was reverse transcribed with a Transcriptor First Strand cDNA Synthesis Kit (Roche Applied Sciences, Indianapolis, IN). Quantitative RT-PCR was performed using Applied Biosystems pre-designed TaqMan Gene Expression Assays for rats and TaqMan® Fast Advanced Master Mix. PCR was performed in the QuantStudio 6 Flex Real-Time System (Applied Biosystems, Waltham, MA). Target gene expression levels were determined by the comparative 2∧-(delta C(T)) quantification method using *Gapdh* as the reference gene, which was previously established to be a suitable reference gene for the anterior pituitary tissue (27). Applied Biosystems predesigned TaqMan Gene Expression Assays were used: *Amhr2* (Rn00513843_m1), *B3galt2* (Rn01759212_m1), *Caly* (Rn01502080_m1), *Cga* (Rn01440184_m1), *Cited1* (Rn06396495_g1), *Cgref1* (Rn00594626_m1), *Fshb* (Rn01484594_m1), *Gal* (Rn00583681_m1), *Gapdh* (Rn01462662_g1), *Gata2* (Rn00583735_m1), *Gfap* (Rn01253033_m1), *Gh1* (Rn01495894_g1), *Kcnmb4* (Rn00576685_m1), *Klk1* (Rn00824646_m1), *Lhb* (Rn00563443_g1), *Lhx3* (Rn00521923_m1), *Lhx4* (Rn01491103_m1), *Pitx3* (Rn01410832_g1), *Pomc* (Rn00595020_m1), *Prl* (Rn00561791_m1), *Pvalb* (Rn00574541_m1), *S100b* (Rn04219408_m1), *S100g* (Rn00560940_m1), *Sec14l3* (Rn01443243_m1), *Sez6l2* (Rn01509033_m1), *Snap25* (Rn00578534_m1), *Sox2* (Rn01286286_g1), *Stmn3* (Rn00694410_g1), *Tmem130* (Rn01412811_m1), and *Tshb* (Rn01534458_g1).

### Immunocytochemistry

For immunocytochemical analysis, 50,000/well freshly dispersed anterior pituitary cells were plated on poly-D-lysine-coated 8-well multi-test slides (MP Biomedicals, Aurora, OH) and bathed in medium 199 containing Earle's salts, sodium bicarbonate supplemented with 10% heat-inactivated horse serum, penicillin (100 units/ml), and streptomycin (100 μg/ml) and cultured overnight. In the morning, cells were washed with PBS two times, fixed with 4% formaldehyde solution (Thermo Scientific, Rockford, IL, USA) for 10 min at room temperature and washed three times with PBS. For immunostaining, pituitary cells were incubated with primary mouse anti-S100B (1:500), rabbit anti-GH (1:250 and 1:500), guinea pig anti-GH (1:500), guinea pig anti-PRL (1:1,000 and 1:2,000), rabbit anti-SOX2 (1:500), rabbit anti-GATA2 (1:500), rabbit anti-ALDH1A1 (1:400), or guinea pig anti-ACTH (1:1,000) antibodies, followed by application of a corresponding secondary antibody (Alexa Fluor 488 donkey anti-mouse, Alexa Fluor 568 donkey anti-rabbit, Alexa Fluor 488 goat anti-rabbit, or Alexa Fluor 568 goat anti-guinea pig; all in 1:1,000 dilution). In single immunostaining for S100B, cells were incubated with anti-S100B antibody overnight at 4°C, followed by incubation with the secondary antibody for 30 min at room temperature. Double immunostaining was done in several combinations: rabbit anti-SOX2 followed by mouse anti-S100B or guinea pig anti-ACTH; rabbit anti-GATA2, followed by guinea pig anti-GH; rabbit anti-GH followed by guinea pig anti-PRL; and rabbit anti-ALDH1A1 followed by guinea pig anti-PRL or mouse anti-S100B. For double immunostaining, pituitary cells were incubated with the first primary antibody overnight at 4°C. After the subsequent incubation with the corresponding secondary antibody for 30 min at room temperature, cells were incubated with the second primary antibody overnight at 4°C, followed by incubation with the secondary corresponding antibody for 30 min at room temperature. All antibodies were diluted in staining PBS solution containing 0.2% saponin and 0.5% BSA. Every step of the immunostaining protocol was followed by washing cells three times with PBS. Cells were mounted with Fluoromount-G, with 4′,6-diamidino-2-phenylindole (DAPI; Invitrogen, Carlsbad, CA). All images were acquired on an inverted confocal laser-scanning microscope (LSM 780; Carl Zeiss GmbH, Jena, Germany), using a 63× Oil objective. Micrographs were sized, and their brightness and contrast levels adjusted in Fiji. Cells were counted on 12 tile-scan images (3 × 3). All experiments were done with anterior pituitary cells from postpubertal female rats and S100B and ALDH1A1 staining was also analyzed in anterior pituitary cells from postpubertal male rats. Each experiment was done using at least two separate cell preparations from different dates, with six replicates per experiment for treatments and two replicates for negative immunostaining controls. Data are shown as total number of positive cells from all experiments or mean ± SEM values.

### scRNA-seq

#### Library Preparation and Sequencing

Single-cell cDNAs and barcoded sequencing libraries were constructed from the output of the Chromium Single Cell Controller using a Chromium Single Cell 3′ Reagent Kit v2 according to the manufacturer's protocol (10X Genomics). The libraries were quantitated, pooled, and sequenced on an Illumina HiSeq2500 operated in RapidRun mode. The multiplexed libraries were run on two Illumina sequencing RapidRun flow cells. An average of 225 million reads were generated per sample. The read lengths were set to: read 1 = 26 bp and read 2 = 98 bp; thus, the sequencing reads are from the distal end of the 3′UTR, near the polyA-tail.

#### Pre-processing of scRNA-seq Data

Using the NCBI reference genome, many reads for expected pituitary transcripts, including *Prop1, Egr1, Isl1, Gnrhr, Smad4, Gnaq*, and *Fgf9*, among others, were mapped near but outside the annotated 3′ boundary of gene annotations, a phenomenon which has been observed in other recent scRNA-seq studies using the 10X Chromium platform (25, 28, 29). To address this, 3′ annotations were extended by the lesser of 4 kb or half way to the nearest feature in a strand independent manner, so that gene annotations never overlapped. Mitochondrial gene annotations from a similarly extended Ensembl Rnor6 reference were appended to the extended NCBI reference. Extending the reference in this manner resulted in 194 genes originally having no counts shifting to having counts in at least 30 cells, and 1,420 genes with at least a 3-fold increase in counts. Among our four samples, the reads mapped confidently to exonic regions increased by 2.5 ± 1%.

De-multiplexing, alignment to the extended reference transcriptome and UMI collapsing were performed using the Cell Ranger toolkit (version 2.1) provided by 10X Genomics. All male and female replicates were aggregated into a single UMI count matrix using Cell Ranger aggr set to mapped normalization mode. The data discussed in this publication have been deposited in NCBI's Gene Expression Omnibus (30) and are accessible through GEO Series accession number GSE132224 (https://www.ncbi.nlm.nih.gov/geo/query/acc.cgi?acc=GSE132224).

A total of 3,729 and 4,132 GEMs were called as female and male cells using default Cell Ranger settings, with a post-normalization mean reads per cell of 67,509, and a median of 2,652 genes per cell and 8,853 total UMI counts per cell. Expression values *E*_*i, j*_ for gene *i* in cell j were defined as normalized UMI counts, calculated by dividing UMI counts for gene *i* by the total UMI count in cell *j*, to normalize for differences in coverage, then multiplying by the median total UMI count per cell. For visualization and some analysis steps, we computed the log_10_ expression with a pseudocount of 1: log_10_ expression = log_10_(E_i, j_ + 1). For display in figures and tables, we converted some gene names from the NCBI reference symbol to the latest Rat Genome Database (RGD) symbols in two cases: first, gene symbols beginning with LOC or RGD were replaced by a more informative RGD symbol if the NCBI gene ID or RGD ID provided such a name; and second, genes for which the genomic location (chromosome, strand, start and stop position) had an exact match between the NCBI reference and the latest RGD annotation, and the RGD symbol was different from the NCBI gene name. This procedure yielded replacement of 1,454 gene symbols, of which only a subset is displayed in our tables. The RGD list of gene annotations file GENES_RAT.txt was downloaded from ftp://ftp.rgd.mcw.edu/pub/data_release/ on 1/24/2019.

#### Dimensionality Reduction

**Figure 1:**
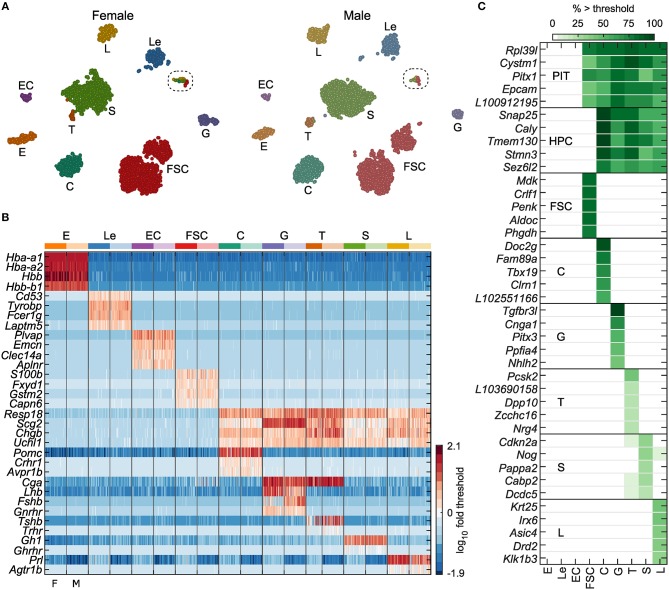
Identification of anterior pituitary cell types. **(A)** tSNE map showing identified cell types for both female and male cells: erythrocytes (E), leukocytes (Le), endothelial cells (EC), folliculostellate cells (FSC), corticotrophs (C), gonadotrophs (G), thyrotrophs (T), somatotrophs (S), and lactotrophs (L). The results of marker-based classification, performed separately per sex, align very closely with the independent clustering done by tSNE. Circled, cells expressing cell cycle genes (see Figure 9 and Table S3). **(B)** Expression of genes used for cell type classification in a random subsample of 50 cells per sex for each cell type, shown as log_10_ fold change relative to gene threshold (see Materials and Methods). Columns alternate between female and male cells (F, M; bottom). **(C)** Selected genes identified as specific markers or cell type-dominant genes, excluding classification genes (see Materials and Methods, Table 1). PIT – pituitary cells, comprising FSC and HPC. The gene name prefix LOC was shortened to L. Cell types E, Le, EC, and FSC were defined by expression > threshold of at least two of the four marker genes indicated per group. Hormone-producing cells (HPC) comprised C, G, T, S, and L, all of which expressed at least two of *Resp18, Scg2, Chgb*, and *Uchl1*. Specific HPC types expressed additional genes as follows: C, at least one of *Pomc, Crhr1*, or *Avpr1b*; G, at least two of *Cga, Lhb, Gnrhr*, or *Fshb*; T, at least two of *Cga, Tshb*, or *Trhr*; S, *Gh1* or *Ghrhr*; L, *Prl* or *Agtr1b*.

**Figure 2:**
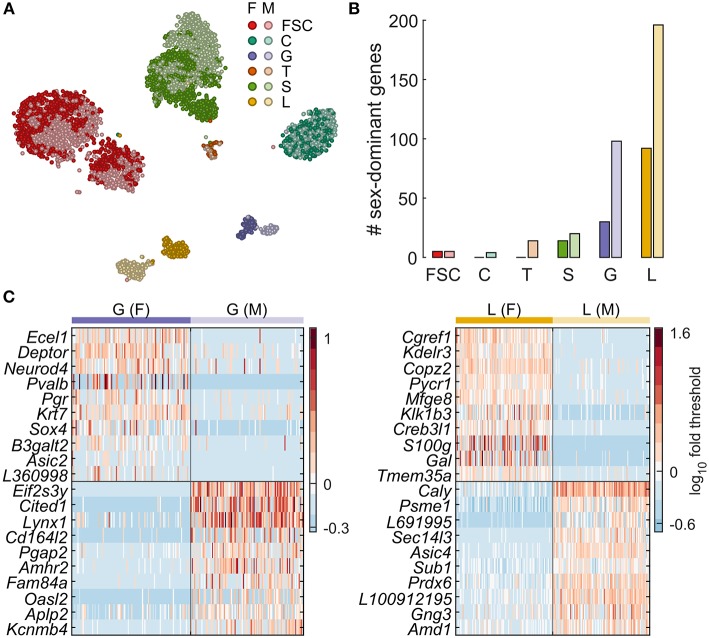
Sexually dimorphic gene expression underlies lactotroph and gonadotroph subclustering. **(A)** Sex-specific clustering of pituitary cells shown as a tSNE plot. For a given cell type, females are indicated with a darker color. **(B)** Number of genes identified as dominantly expressed per sex for each cell type. **(C)** Expression of genes with the highest difference in % cells expressing > threshold between sexes for G and L. A random subsample of 100 female (left of vertical line) and 100 male cells (right of vertical line) are shown. The top 10 genes for females are shown above the horizontal black line, and those for males below. Sex-dominant and sex-specific genes identified by differential expression analysis are listed in Table 3. F, female; M, male. The gene name prefix LOC was shortened to L.

**Figure 3:**
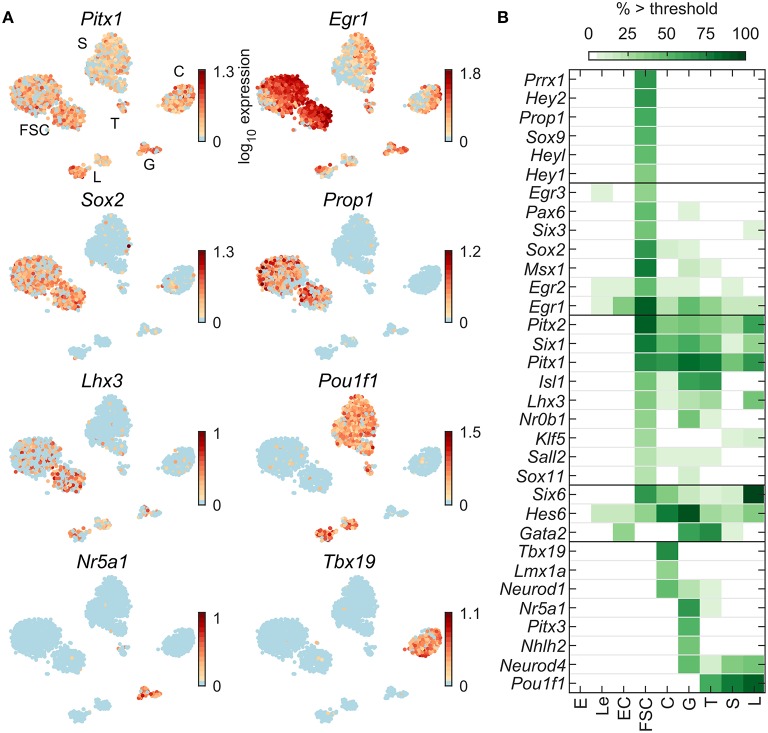
Expression of pituitary development/differentiation genes. **(A)** tSNE map of expression for selected genes common to all pituitary cells (*Pitx1*), FSC-dominant (*Egr1, Sox2*), FSC-specific (*Prop1*), co-expressed by FSC and some HPC (*Lhx3*), and HPC type-specific (C—*Tbx19*; T, S, L—*Pou1f1*; G—*Nr5a1*). **(B)** Percentage of cells per type expressing selected genes above threshold. For this and Figures 4–8, genes are grouped into five categories based on their cell type-dominant or -specific expression. From top to bottom: FSC-specific, FSC-dominant, dominantly expressed in both FSC and HPC relative to non-pituitary cells, HPC-dominant (in at least one HPC type), and HPC-specific. From a manually selected list of developmental genes, 64 were expressed in at least 5% of cells in at least one cell type (Table 2), and all 33 pituitary-dominant genes are shown.

**Figure 4:**
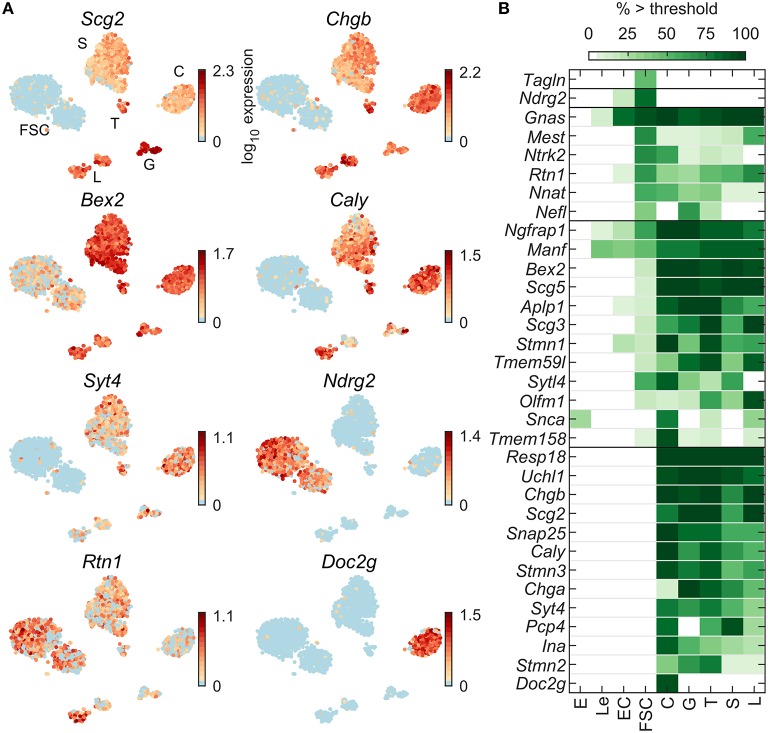
Expression of neuroendocrine marker genes. **(A)** tSNE map showing expression of select genes: granins (*Scg2, Chgb*), upstream modulators of receptor signaling (*Bex2, Caly*), downstream regulators of receptor signaling (*Ndrg2*), synaptotagmins (*Syt4*) and neuroendocrine cell vesicular trafficking (*Rtn1, Doc2g*). **(B)** Percentage of cells per type expressing select genes above threshold. From a manually selected list of neuroendocrine markers, 99 were expressed in at least 5% of cells in at least one cell type (Table 2) and 70 were pituitary dominant (Table S1). The 33 most highly expressed of these are shown.

**Figure 5:**
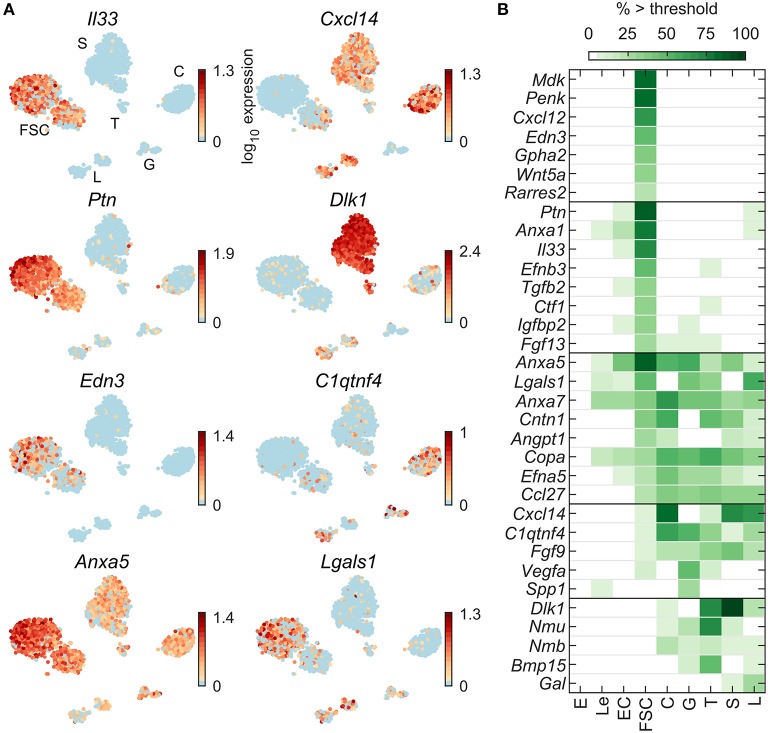
Expression of endogenous ligand genes. **(A)** tSNE map showing expression of select genes from the following families: cytokines (*Il33*), chemokines (*Cxcl14*), neuronal ligands (*Ptn, Dlk1*), ligands for G-protein coupled receptors (*Edn3, C1qtnf4*), and ligands that use other signaling pathways (*Anxa5, Lgals1*). **(B)** Percentage of cells per type expressing select genes above threshold. From the list of HGNC endogenous ligands gene family members, 94 were expressed in at least 5% of cells in at least one cell type (Table 2) and 44 were pituitary dominant (Table S2). The 33 most highly expressed of these are shown.

**Figure 6:**
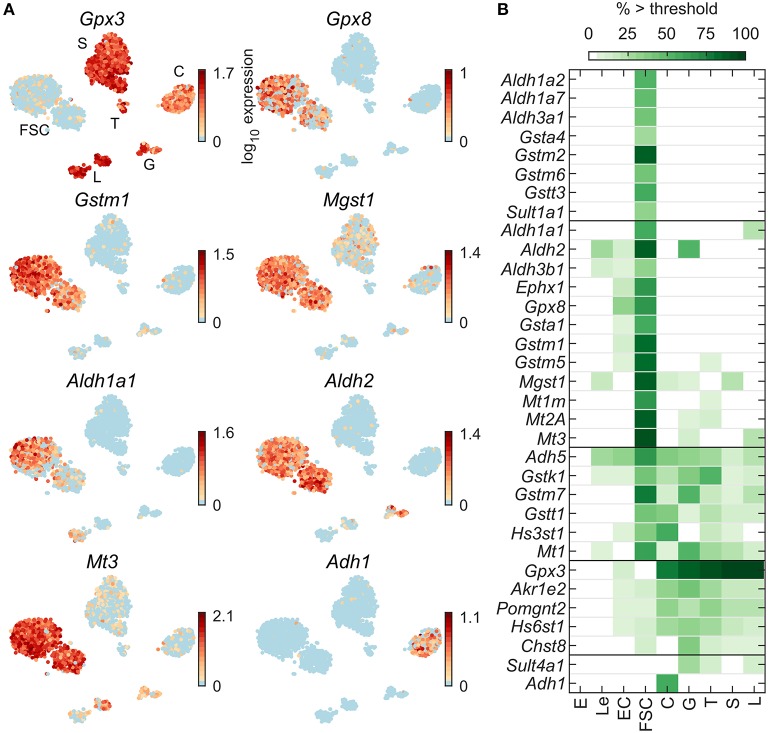
Expression of detoxification enzyme genes. **(A)** tSNE map showing expression of select genes from the following families: selenoproteins (*Gpx3, Gpx8*), glutathione S-transferases (*Gstm1, Mgst1*), aldehyde dehydrogenases (*Aldh1a1, Aldh2*), metallothioneins (*Mt3*), and alcohol dehydrogenases (*Adh1*). **(B)** Percentage of cells per type expressing select genes above threshold. From a manually selected list of detoxification enzyme genes, 79 were expressed in at least 5% of cells in at least one cell type (Table 2) and all 33 pituitary-dominant genes are shown.

**Figure 7:**
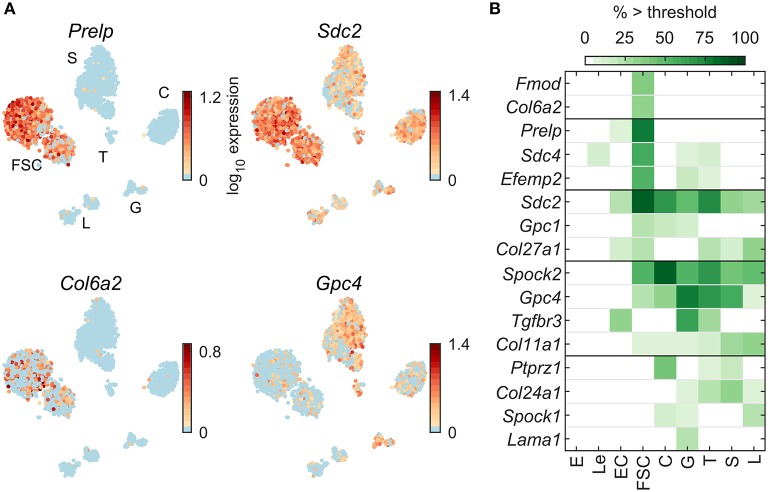
Expression of genes encoding extracellular matrix protein. **(A)** tSNE map showing expression of select members of extracellular matrix gene families: small leucine-rich proteoglycans (*Prelp)*, syndecans (*Sdc2*), collagens (*Col6a2*), and glypicans (*Gpc4*). **(B)** Percentage of cells per type expressing select extracellular matrix genes above threshold. From a manually selected list of extracellular matrix genes, including the genes annotated to the Gene Ontology term GO:0005201—Extracellular Matrix Structural Constituent, 53 were expressed in at least 5% of cells in at least one cell type (Table 2) and all 16 pituitary-dominant genes are shown.

**Figure 8:**
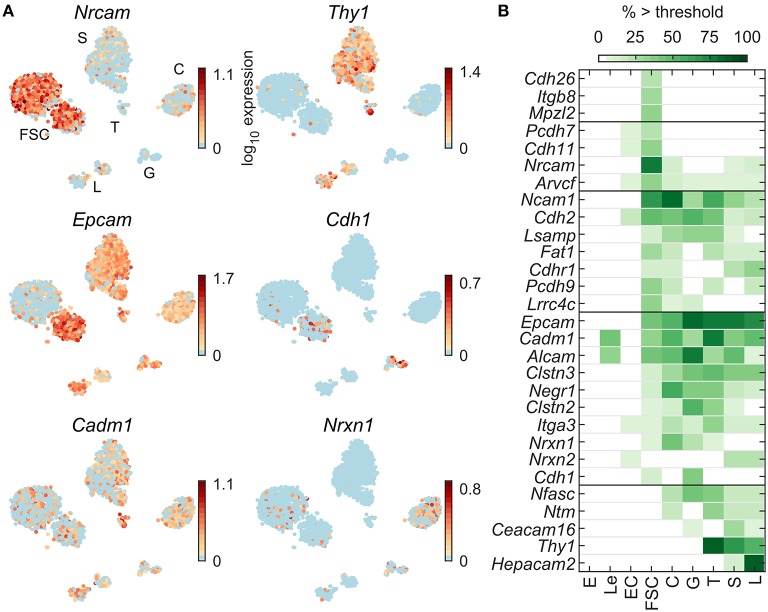
Expression of genes encoding cell adhesion molecules. **(A)** tSNE map showing expression of select members of cell adhesion gene families: immunoglobulin-like (*Nrcam, Thy1, Cadm1*), cadherins (*Cdh1*), and type-I membrane proteins containing EGF-like domains (*Epcam, Nrxn1*). **(B)** Percentage of cells per type expressing cell adhesion genes above threshold. From a manually selected list of cell adhesion molecule genes, including the list of HGNC cell adhesion molecule gene family members, 87 were expressed in at least 5% of cells in at least one cell type (Table 2) and all 29 pituitary-dominant genes are shown.

**Figure 9:**
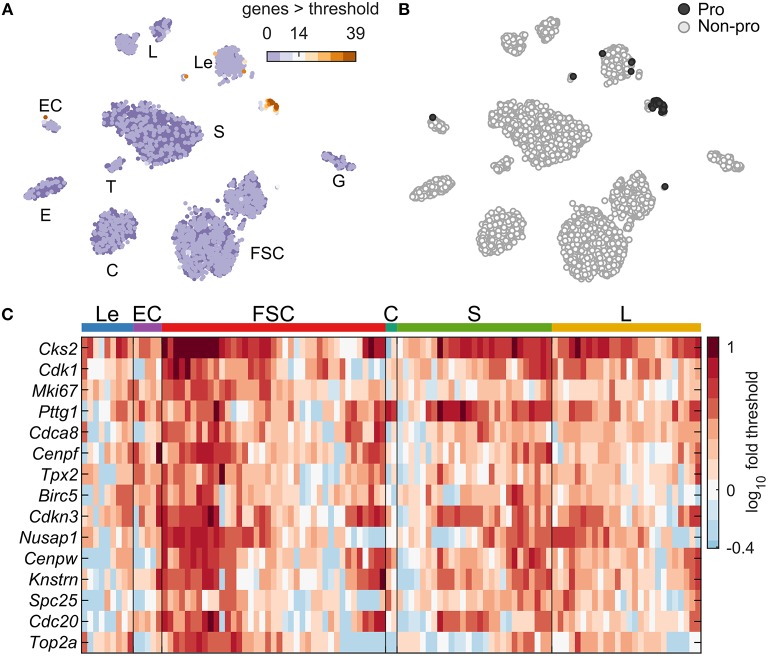
Expression of cell cycle genes. **(A)** tSNE map including cells of both sexes showing the cell cycle score, defined as the number of cell cycle marker genes with expression > threshold per cell. A set of 39 cell cycle marker genes was identified by an iterative classification method (Table S3, Materials and Methods). **(B)** tSNE map showing the identity of cells defined as proliferating (Pro, dark gray; 108 cells with ≥14 cell cycle marker genes expressed) or not proliferating (Non-pro, white). **(C)** Expression heatmap showing the top 15 expressed cell cycle marker genes, and the cell type identity of each proliferating cell. The 108 cells were divided among Le (9 cells), EC (5 cells), FSC (39 cells), C (2 cell), S (27 cells), and L (26 cells). No proliferating cells were observed in E, G, and T.

Principle component analysis (PCA) was used for gene-space dimensionality reduction prior to downstream computation of tSNE maps, hierarchical clustering, and identification of cells' k-nearest neighbors. PCA was performed using Matlab's svds function on log_10_ expression values of the top 1,000 highly variable genes that were expressed in at least 30 cells (Matlab R2018b, The Mathworks, Natick MA, 2018b). Highly variable genes were identified with an in-house version of the method used in the 10X Genomics Cell Ranger software (31). Genes were binned into 20 bins based on mean normalized counts. Dispersion was estimated for each gene as ϕ = *(s*^2^*-m)/m*^2^, where *m* is the sample mean and *s*^2^ is the sample variance. Dispersion values were normalized within each bin by subtracting the bin median then dividing by the bin median absolute deviation, and genes with the top normalized dispersions were selected. The log_10_ expression values of the selected genes were centered and scaled by standard deviation, and resulting standardized values were clamped to [-10,10] to reduce the effect of genes highly expressed only in small subpopulations of cells as is done in Seurat (http://www.satijalab.org/seurat.html). A final reduction was done by retaining only principle components (PCs) that were found to be significant by a permutation test, similarly to previously described methods (32). Briefly, UMI counts were shuffled among cells for each gene independently, maintaining gene count distributions but destroying cell-wise correlations, and singular values were computed for the resulting count matrix. This was repeated 100 times to generate a null distribution of maximum singular values, into which the observed singular values could be ranked. The number of significant components was taken as the number of singular values that were larger than the maximum of the null distribution. This resulted in 26 (31) PCs for female (male) cells for use in cell type classification, 32 PCs for generation of the tSNE plot of all cleaned cells, 31 PCs for the tSNE plot of pituitary cells only. Computation of tSNE maps was done using the tsne function in the Matlab Statistics and Machine Learning Toolbox (R2018b), with perplexity of 40 and 2,000 iterations. A separate tSNE map was computed for all cells (Figure 9), all male and female cells separately (Figure 1 and Figure S2), and all pituitary cells (Figures 2–8).

#### Cell Type Classification and Quality Control

Initial inspection of gene expression profiles using the Loupe Browser (10X Genomics) indicated that 9 major cell types and at least three small populations of contaminant cell types could be identified using known marker genes. The use of marker genes was impaired by the fact that several highly expressed transcripts had non-specific expression at lower levels in cell types known not to express them, such as *Gh1* in erythrocytes and *Hbb* in somatotrophs. To overcome this, we computed an optimal log_10_ expression threshold by Otsu's method (33) for each gene, allowing the classification of cells as expressing the gene (log_10_ expression ≥ threshold) or not (log_10_ expression < threshold). The optimal threshold is the value which maximizes the between-class variance of these two classes (33). We computed a histogram of each gene's log_10_ expression values with 256 bins, and bin edges were taken as the possible threshold levels. The between-class variance, $\sigma_{\text{B}}^{2}$, was computed recursively across all 256 threshold candidates, and the gene threshold was defined as the threshold value maximizing $\sigma_{\text{B}}^{2}$. For genes with unimodal distributions and dropouts, this approach leads to an underestimate of the percentage of cells expressing a given gene. For consistency, we applied this thresholding algorithm to all genes for cell-type classification, counting of percentages of cells expressing genes, and visualization of expression relative to the gene-wise threshold.

Pericytes, melanotrophs, and posterior pituitary cells could be identified using marker genes, but were excluded due to their small number and because the intermediate and posterior lobes were removed during dissection. Pericytes were identified by expression > threshold in at least two of *Col1a1, Col1a3, Dcn*, or *Lum* (48 cells) (34, 35). Melanotroph cells from the intermediate lobe were identified as expressing at least two of *Pomc, Pcsk2*, or *Pax7* (36) (58 cells). The transcription factor gene *Tbx19* was also expressed in these cells, as expected for melanotrophs (37). GEMs containing tissue putatively of posterior pituitary origin (possibly pituicytes and neurosecretory oxytocin or vasopressin neuron terminals) were identified by the presence of at least two of *Lhx2* (38), *Nkx2-1*, also known as TTF-1 (39), *Gfap, S100b, Avp*, or *Oxt* (56 cells).

For the remaining cells, cell type classification was performed hierarchically and independently for cells of each sex. First, E, Le, EC, FSC, and HPC were identified. Within the set of HPC, we then identified C, G, T, S, and L. To overcome the problem of dropouts (zero expression values) for known marker genes and to improve the robustness of classification, a set of several marker genes was used, for which expression of subsets of these markers is specific to a given cell type. For some cell types, an initial differential expression analysis between cell types classified with known makers was performed using the Loupe Browser to identify a larger set of marker genes. Expression > threshold in sufficiently many marker genes was taken to indicate cell type identity. The following classification criteria were used: E, expression > threshold for at least two of *Hbb, Hbb-b1, Hba-a1*, or *Hba-a2*; Le, two of *Cd53, Tyrobp, Fcer1g*, or *Laptm5*; EC, two of *Plvap, Emcn, Clec14a*, or *Aplnr*; FSC, two of *S100b, Fxyd1, Gstm2*, or *Capn6*; HPC, two of *Resp18, Uchl1, Chgb*, or *Scg2*; C, two of *Pomc, Crhr1*, or *Avpr1b*; G, two of *Cga, Lhb, Gnrhr*, or *Fshb*; T, two of *Cga, Tshb*, or *Trhr*; S, one of *Gh1* or *Ghrhr*; L, one of *Prl* or *Agtr1b*.

The fraction of UMI counts mapped to mitochondrial transcripts (f_MT_) was measured in classified cells, and cells with high f_MT_ were excluded as potentially damaged cells (Figure S1A). The distribution of f_MT_ differed among cell types, so within each cell type cells with f_MT_ higher than 2 standard deviations above the mean were excluded, for a total of 278 cells. Similarly, we excluded outliers in the distributions of genes per GEM and total UMI counts per GEM in a cell-type specific manner (Figure S1B). We excluded cells in the highest and lowest 1.5% of the distribution, for a total of 305 cells. Thirty nine cells had both high f_MT_ and were gene or UMI outliers.

The marker-based classification scheme could not classify 249 cells and identified 192 ambiguous cells satisfying more than one cell type criterion. Nearest-neighbor imputation was used to try to assign a cell type to unclassified cells. For each unclassified cell, its 30 nearest neighbors of the same sex were identified in PC space using Matlab's knnsearch function. Unclassified cells were assigned the cell type most frequent among their neighbors. Imputation succeeded for 225 of the unclassified cells. Cells remained unclassified if the most frequent neighbor class was ambiguous or unclassified (24 cells).

Ambiguous cells could be due to GEMs containing more than one different cell type (multiplets) or shortcomings of the marker-based classification scheme. Most multiplets were expected to be doublets, so we computationally identified doublet GEMs using an algorithm similar to the recently reported methods DoubletDetection (https://github.com/JonathanShor/DoubletDetection), DoubletFinder (40) and Scrublet (41). We assumed that heterotypic doublets should cluster separately in gene space from the cell types that comprise them and that these clusters should be detectable if doublets appear in sufficient numbers. We boosted the set of true cells with 30% additional synthetic doublet cells created from randomly selected pairs of true cells, with replacement, for male and female cells separately (Figures S2A,B). Synthetic cell UMI counts were randomly subsampled from both parent cells to obtain a library size equal to the larger of the two parents. PCA dimensionality reduction was then performed on the boosted dataset. A k-nearest neighbor classification was then used to identify putative doublet cells. For each true cell in the dataset, 30 nearest neighbors were identified and the number of synthetic doublet neighbors were counted. The hypergeometric test was used to compute the probability of observing at least this many synthetic doublets in the group of 30 neighbors, given the known total numbers of true and synthetic cells (using Matlab's hygecdf function with the “upper” option). The *P*-values were then adjusted for multiple testing (one test per true cell) using Benjamini-Hochberg correction (42), and a GEM was deemed a doublet if adjusted-*P* < 0.01. The process was repeated 10 times and doublets identified in any of the repetitions were taken as the final set of cells identified as doublets. The value of boost rate (30%) and number of nearest neighbors (nKNN = 30) were chosen empirically to be where the median number of doublets identified per repetition plateaued (Figure S2C). The algorithm identified 146 doublet cells, in line with the expected proportion of roughly 2% for each of our lanes of 2,000 cells (31). Some of these doublets were also identified as cells to be removed for other reasons already mentioned. In total, 87 of the 192 ambiguous cells and 2 of the 24 unclassified cells from marker-based classification were also identified as doublets. Interestingly, only 3 of 278 cells with high f_MT_ and 12 of 305 outlier cells, respectively, were also identified as doublets, suggesting that outliers in mitochondrial fraction, genes per cell, or counts per cell are not a reliable method for identifying doublets in this dataset. Finally, gene thresholds for further analysis were computed using the final set of cells with cells of both sexes pooled together.

#### Differential Expression Testing and Definition of Dominant and Specific Genes

To identify differentially expressed genes, we tested both for changes in expression and changes in proportion of cells expressing > threshold. Tests for changes in expression were performed for each gene using the non-parametric Kruskal-Wallis test (Matlab's kruskalwallis function) followed by multiple comparisons between all pairs of cell types, with Bonferroni correction (Matlab's multcompare function). Similarly, we used Pearson's Chi-square test for independence for the proportion of cells expressing a gene > threshold, followed by pairwise *Z*-tests for the difference in proportions for each pair of cell types. The ANOVA *P*-value, Pearson's Chi-square *P*-value, and the pairwise contrast *P*-values were corrected for multiple comparisons to account for the number of genes tested using the Benjamini-Hochberg correction (42). We considered a gene as significantly upregulated if the expression test or proportion test passed (adjusted ANOVA *P*-value and every *P*-value for the relevant pairwise contrasts were <0.001), and if there was an accompanying increase in mean expression or proportion of cells expressing the gene in the cell type of interest, relative to all other cell types being compared. For tests of differential expression between sexes, the Wilcoxon Rank Sum test was used (Matlab's ranksum function), followed by multiple correction for the number of genes tested.

To further identify genes of interest from the set of significantly upregulated genes, we considered only genes with at least 20% of cells expressing in a cell type of interest to avoid genes only expressed in a small subpopulation. We defined genes as *dominantly expressed* in a cell type if in addition they had at least 3-fold greater mean expression or 30% higher proportion of cells expressing > threshold in that cell type, relative to all other cell types of interest. We defined *specific marker genes* as dominantly expressed genes for which the % of cells expressing that gene was below 5% in all other cell types. For groups of cell types (PIT and HPC), the test for dominant or specific expression was satisfied for every pairwise combination of cell types within the group, relative to cell types outside the group.

#### Identification of Cell Cycle Genes and Proliferating Cells

Initial investigation using the Loupe Browser showed a small group of cells expressing mitotic cell cycle markers. Beginning with a curated list of cell cycle genes (43), we identified proliferating cells and a set of cell cycle marker genes in our dataset using an iterative process. A cell cycle score was defined per cell as the number of genes in the list of markers that were expressed > threshold in that cell. Otsu's optimal threshold was then computed on the distribution of cell cycle scores for all cells, and cells whose score exceeded the threshold were classified as candidate proliferating cells. Next, genes with significantly upregulated expression (Wilcoxon Rank Sum test, adjusted-*P* < 0.001) in at least 50% of these cells, but <5% of the remaining cells, and that were also annotated with Gene Ontology term “mitotic cell cycle” (GO:0000278), were selected as the updated list of cycle marker genes. This updated list was then used for the next round of cell cycle score computation and cell classification. The process was repeated until the list of mitotic cell cycle marker genes and the cell classification stabilized. A final list of 39 genes was identified (Table S3) with a final gene-count threshold of 14 genes expressed, which identified 108 cycling cells.

## Results

### Cell Populations in the Anterior Pituitary Gland and Their Marker Genes

Cells were dispersed using anterior pituitaries from four postpubertal female and male rats. scRNA-seq was done using four lanes on the 10X Genomics Chromium Single Cell Controller (10X Genomics, Pleasanton, CA), providing two technical replicates for each sex. A total of 7,861 cells were recovered: 1,868 and 1,861 for diestrus females, and 2,047 and 2,085 per lane for males. Quality control removed 965 cells, including 60 melanotrophs (see Materials and Methods), leaving 6,896 cells for further analysis. There were no significant differences in expression patterns between the two technical replicates for each sex, so the samples were pooled resulting in 3,334 female cells and 3,562 male cells for further analysis.

The t-distributed stochastic neighbor embedding (tSNE) maps in Figure 1A show numerous clusters similarly in each sex. Nine cell types were identified based on expression of known marker genes, as described in the Figure 1 legend and Materials and Methods. The log_10_ expression of marker genes used in classification is shown in Figure 1B. The marker gene-based classification gave cell labeling consistent with the tSNE clusters. Single clusters accounted for most cell types, while most notably FSC showed two distinct clusters. The final set of classified cells consisted of E (364), Le (556), EC (137), FSC (2,327), C (800), G (245), T (89), S (1,888), and L (490).

To study gene expression in the identified cell populations, we used a two-step differential expression analysis to define cell type-dominant genes and cell type-specific genes. Cell type-dominant genes were defined as genes significantly upregulated in a cell type or group of cell types of interest relative to remaining cell types (for details, see Materials and Methods and Table 1). Because these dominant genes could also be expressed at lower levels by some other cell types, we also defined a subset of dominant genes as cell type-specific markers if they were expressed in <5% of cells in every other cell type. This analysis revealed 60 genes dominantly co-expressed among all pituitary cells, distinguishing them from EC, Le, and E. Similarly, we found 69 genes dominantly co-expressed among all HPC, distinguishing them from FSC, EC, Le, and E. We report 208 FSC-dominant genes, and individual HPC types C, G, L, S, and T, had 67, 72, 37, 16, and 29 dominant genes, respectively (Table 1). The top five specific marker genes for each of these cell groups are shown in Figure 1C.

**Table 1:** Genes dominantly and specifically expressed in anterior pituitary cell types.

**Pituitary cells:** *Acyp2, Arl3, **Arl6**, Casc4, **Ccl27**, Chchd10, **Cldn9**, Cpe, **Cyb561**, **Cystm1**, **Dzip1**, Eif4a2, **Epcam**, Fam162a, **Fbxo44**, **Fuz**, **Gnao1**, **Gprasp1**, **Hap1**, Klhdc8b, **LOC100912195**, **MGC109340**, Maged1, Maged2, Map1b, Mdh1, **Minar2**, **Morn2**, **Ncam1**, Ngfrap1, **Nudt11**, Pigp, **Pitx1**, **Pitx2**, Pja1, **Pnma8b**, Prrt1, Psip1, **Rab25**, **Rab36**, Rogdi, **Rpl39l**, **Shisa4**, **Smim22**, Spa17, Spint2, **Spock2**, **Tceal3**, Tceal9, Tctex1d2, Tmem107, Tspan3, Tspyl4, **Tubb4a**, Tusc3, Wdr6, **Zcchc12**, **Zcchc18**, Zfp612, Zwint*.
**Hormone-producing cells:** ***Actl6b**, **Ankrd37**, **Ap3b2**, Aplp1, **Asphd1**, Atp5if1, Atp6v1b2, Bcat1, Bex2, Cacna2d1, **Caly**, **Camk2b**, Cbarp, **Celf3**, Chchd6, **Chgb**, Ckmt1b, Cnih2, Crb3, Fam171b, Fam92b, **Fbxl16**, Fkbp11, **Gabrb3**, **Gdap1l1**, Gng3, Gpx3, Hist2h2aa2, **Hist2h2aa3**, **Insm1**, Kif1a, Kif5c, **Krtcap3**, **LOC100362965**, **Lrrc73**, **Mapk8ip2**, Mllt11, Nalcn, **Nap1l5**, Pld3, Ptprn, **Ptprn2**, Rab3a, Rasd1, **Resp18**, **Rltpr**, **Rundc3a**, **Scg2**, Scg3, Scg5, **Scn3b**, Sec11c, Sec61b, Sec61g, Serpini1, **Sez6l2**, Sgsm1, **Snap25**, **Snap91**, **Sprn**, Srp14, Ssr4, **Stmn3**, **Syp**, **Syt4**, **Syt7**, **Tmem130**, **Uchl1**, **Unc80***.
**Folliculostellate cells:** *Abi3bp, **Ackr4**, **Acss1**, **Acy3**, Adamts9, Aldh1a1, **Aldh1a2**, **Aldh1a7**, Aldh2, **Aldh3a1**, Aldh3b1, **Aldoc**, Anxa1, Arhgap8, Arvcf, Bcl2, **Bdh2**, C1qtnf5, C1r, **C1s**, Calml4, **Capn6**, **Ccdc153**, Ccdc60, Ccdc80, Cd24, Cdh11, **Cdh26**, Cdk6, Ces1d, **Cldn10**, **Clic6**, **Col6a2**, **Cox6b2**, **Crlf1**, Crot, **Cryab**, **Crym**, Ctf1, **Cxcl12**, Cyb5r2, Cyb5r3, Cybrd1, Cyp7b1, Dapl1, Dhrs4, **Edn3**, Efemp2, Efhd1, Efnb3, Egr1, Egr2, Egr3, Ephx1, **Etv4**, **Fam107a**, Fam181b, Fgf13, **Fgfr3**, **Fhad1**, **Fibin**, **Fmo1**, **Fmod**, Fosb, **Foxj1**, **Fxyd1**, Fzd1, Galm, Galnt18, Gas1, Gmpr, **Gpha2**, Gpm6b, **Gpr37l1**, Gpx8, **Grifin**, Gsg1l, Gsn, Gsta1, **Gsta4**, Gstm1, **Gstm2**, Gstm5, **Gstm6**, **Gstt3**, Gucy1a3, **Gxylt2**, **H1foo**, **Hey1**, **Hey2**, **Heyl**, **Hsd11b1**, **Hspb2**, Hspb8, Hunk, Id4, Igfbp2, **Il20rb**, Il33, **Itgb8**, Kctd1, Klhl36, LOC100909835, Lcn2, Lingo2, **Lpar3**, Lrp1, **Lypd1**, Mageh1, **Maob**, **Marveld3**, Mdfic, **Mdk**, Metrn, Mfap2, Mgst1, Mocs1, Mpp6, **Mpzl2**, Msx1, Mt1m, Mt2A, Mt3, Mxra8, **Myh7**, Myl9, Ndrg2, Nfia, Nfix, **Nkain4**, Notch2, Npnt, Nqo1, Nrcam, **Nrgn**, **Nwd1**, Oat, **Olfml1**, **P2rx2**, Paqr6, Patz1, Pax6, Pcdh7, Pde1c, Pdlim4, **Pdpn**, **Penk**, **Phgdh**, Pla2g7, Plat, **Plch1**, Plekhb1, Pmp22, Prelp, **Prop1**, Prps2, **Prrx1**, **Psat1**, Ptch1, Ptn, Pxmp2, RGD1305645, **RGD1306233**, **RGD1564162**, Rab34, **Rarres2**, **Rbp1**, Rbpms, Rdh10, **Rfx4**, **Rgma**, Rnd3, S100a1, S100a11, S100a6, **S100b**, Sdc4, **Serping1**, **Sfxn5**, Shroom3, Six3, **Slc16a11**, Slc1a3, **Slc20a2**, Slc6a8, **Slc9a3r1**, **Slit2**, **Smo**, Smpdl3a, **Sod3**, Sox2, **Sox9**, **Spats2l**, Spry2, Sspn, **Sult1a1**, **Tagln**, Tgfb2, Timp1, Tmem17, Tmtc2, Tst, Ttyh1, Tubb2a, Tuft1, Vangl2, **Wnt5a**, Zfp423*.
**Corticotrophs:** *Adcy2, **Adh1**, **Angptl8**, Apol9a, Armcx2, Armcx3, Armcx6, **Avpr1b**, Cacng2, Camk1, **Ccdc155**, Cdo1, Celf4, Cenph, Chkb, **Chrna1**, **Clrn1**, **Crhr1**, Dgkz, Dmrtc1a, **Doc2g**, Dsp, Dusp26, Elavl2, **Fam89a**, Galnt14, Gap43, Ggt7, Hdac11, **Hspb3**, **Il22**, Ina, **LOC102551166**, **Lmx1a**, Ly6h, Mdm1, Mmd, Mrap2, Mxra7, N4bp2l1, Neurod1, Nudt8, Plcd4, **Pomc**, Pqlc3, Prr15l, Ptprz1, **RGD1305464**, Rab3b, **Rab6b**, **Scube2**, Slc35g2, Smpd3, Snca, Sncb, Stxbp1, Syt13, Tapbpl, **Tbx19**, Tmem158, Tmem38a, Tmem50b, **Trdn**, Trim54, Tstd1, Ugp2, Zfp57*.
**Gonadotrophs:** ***Aif1l**, Amhr2, Apip, Appl2, Ar, B3galt2, Camta1, **Cd164l2**, Cdh1, **Chrna4**, Cited1, **Cnga1**, Ctxn1, **Defb24**, **Dusp15**, Enho, Fkbp1b, Fndc10, Foxp2, **Fshb**, Galc, Gjd2, **Gnrhr**, Hoga1, **Icam5**, Igdcc4, **Ihh**, Inha, Kcna4, Kcnh6, Kcnk3, **Kcnmb4**, Krt7, LOC100909954, **LOC102557071**, LOC684395, **LOC689757**, **Lad1**, **Lama1**, **Lhb**, Lhpp, Lrrc3b, **MGC93861**, **Nhlh2**, Nr5a1, **Ntrk3**, Obsl1, Otof, Pcsk5, Pdcl2, Pdyn, Pgr15l, **Pitx3**, **Ppfia4**, Prr15, Prr23d2, Pvalb, Rapgefl1, Rfx3, Rgs4, Rida, **Rnf183**, Sept3, Slc35g1, Spp1, Tesc, Tgfbr3, **Tgfbr3l**, Tmem206, Uba6, **Vash2**, Vegfa*.
**Somatotrophs:** *Arhgap36, Cabp2, Cdkn2a, Dcdc5, **Gh1**, **Ghrhr**, Htatsf1, Krt79, Nog, **Pappa2**, Ramp1, Rbp4, Slc7a5, Srxn1, Ttc14, Wfdc1*.
**Lactotrophs:** ***Agtr1b**, **Alk**, Arpp21, **Asic4**, Cgref1, Chl1, Dhx34, **Drd2**, Egfl8, Eno3, Gal, Gpr22, Hepacam2, Insyn1, **Irx6**, Ispd, **Klk1b3**, **Krt25**, LOC102552540, **LOC102555474**, LOC102557241, LOC363337, LOC679711, **LOC685488**, Mlf1, Olfm1, Pde1a, **Pla2g2f**, **Prl**, RGD1562660, Rprm, S100g, **Sec14l3**, Six6, Smc2, **Sostdc1**, Spock1*.
**Thyrotrophs:** *Adam23, Arg1, Atp6ap1l, Bdnf, Bmp15, Cml3, Dio2, Dnah11, **Dpp10**, Fam183b, Gdf11, Gpr158, Grem1, Irs4, **LOC103690158**, Nell1, Nmu, **Nrg4**, Parm1, Pcsk1, **Pcsk2**, Pgm2l1, Prodh, Rxrg, Trhr, Trnp1, **Tshb**, **Zcchc16**, Zfp365*.

*Pituitary cells comprised folliculostellate cells (FSC) and hormone-producing cells (HPC); the latter comprised corticotroph (C), gonadotroph (G), thyrotroph (T), somatotroph (S), and lactotroph (L) cells. Cell type-dominant expression was defined by at least 20% of cells expressing in the cell type of interest and a significant (adjusted-P < 0.001) upregulation by 3-fold higher mean expression or 30% higher proportion of cells expressing > threshold relative to all other cell types, including erythrocytes (E), leukocytes (Le), and endothelial cells (EC). Pituitary cell-dominant and HPC-dominant genes were identified by requiring every cell type comprising the group to satisfy the same criteria. Bold indicates cell type-specific marker genes, which were dominant genes with <5% cells expressing > threshold in all other cell types*.

Inspection of these genes suggested several groups of genes based on function that were enriched in FSC and/or HPC: pituitary development/differentiation genes, neuroendocrine genes, genes listed in the HUGO Gene Nomenclature Committee (HGNC) gene group “endogenous ligands,” genes encoding detoxification enzymes, ECM genes, and cell adhesion molecule genes. These findings motivated the definition of lists of genes for these six gene groups by manual inspection and/or inclusion of Gene Ontology terms and HGNC gene groups, described in detail below. The final list of genes considered for these six categories includes only genes that were expressed in at least 5% of cells in at least one pituitary cell type (Table 2). The remaining FSC- and HPC type-dominant genes included genes encoding transcription factors, plasma membrane receptors, channels, transporters, intracellular signaling molecules, small G proteins, heat shock proteins, cytoskeleton proteins, Tmem family genes, and solute carriers.

**Table 2:** Specific groups of genes expressed in anterior pituitary cells.

**Development/Differentiation:** *Egr1, Egr2, Egr3, Foxl2, Gata2, Hes1, Hes6, Hey1, Hey2, Heyl, Isl1, Klf10, Klf11, Klf12, Klf13, Klf15, Klf16, Klf2, Klf3, Klf4, Klf5, Klf6, Klf7, Klf9, Lhx3, Lhx4, Lmx1a, Msx1, Mta1, Mta2, Mta3, Neurod1, Neurod4, Nhlh2, Nr0b1, Nr5a1, Pax6, Pitx1, Pitx2, Pitx3, Pou1f1, Prop1, Prrx1, Sall1, Sall2, Six1, Six2, Six3, Six4, Six5, Six6, Sox11, Sox12, Sox14, Sox2, Sox30, Sox4, Sox5, Sox6, Sox8, Sox9, Tbx19, Tbx3, Trps1*.
**Neuroendocrine marker genes:** *Apbb1, Aplp1, Aplp2, Atxn10, Bap1, Bex2, Caly, Chga, Chgb, Doc2b, Doc2g, Ephb1, Ephb3, Fgf13, Fuz, Gmfb, Gmfg, Gnas, Gpm6b, Hap1, Ina, Lrrn1, Lrrn2, Lrrn3, Lynx1, Manf, Mest, Metrn, Mtdh, Mxra8, Ndn, Ndrg2, Nedd4, Nefl, Nell2, Ngb, Ngfrap1, Nnat, Npdc1, Nptn, Nptxr, Nrep, Nrg1, Nrgn, Nrsn1, Nrsn2, Nsg1, Nsg2, Ntrk2, Ntrk3, Olfm1, Olfm3, Olfml1, Olfml3, Pcp4, Pcsk1, Pcsk2, Pmp22, Pnma8b, Resp18, Rtn1, Rtn3, Scg2, Scg3, Scg5, Smim3, Snap25, Snap91, Snca, Sncb, Stmn1, Stmn2, Stmn3, Stxbp1, Syp, Syt1, Syt11, Syt12, Syt13, Syt14, Syt16, Syt17, Syt3, Syt4, Syt5, Syt6, Syt7, Syt9, Sytl1, Sytl2, Sytl4, Tagln, Tagln2, Tmem158, Tmem59l, Tubb3, Uchl1, Uchl5, Vgf*.
**Endogenous ligands:** *Agrp, Agt, Angpt1, Anxa1, Anxa10, Anxa11, Anxa2, Anxa3, Anxa4, Anxa5, Anxa6, Anxa7, Apln, App, Artn, Bmp1, Bmp15, Bmp5, Bmp7, C1qtnf4, Calca, Cartpt, Ccl2, Ccl27, Cflar, Cntn1, Cntn2, Cntn3, Copa, Ctf1, Cx3cl1, Cxcl1, Cxcl12, Cxcl14, Cyr61, Dlk1, Edn3, Efna1, Efna2, Efna4, Efna5, Efnb1, Efnb2, Efnb3, Fgf12, Fgf13, Fgf14, Fgf9, Flt3lg, Fst, Gal, Gpha2, Icam1, Icam5, Igf1, Igfbp2, Igfbp4, Igfbp5, Igfbp7, Il22, Il33, Il34, Inha, Inhba, Inhbb, Kiss1, Lgals1, Lgals3bp, Lgals8, Lgals9, Lrrc4b, Mdk, Mif, Nmb, Nmu, Nog, Nppc, Nrtn, Pdyn, Penk, Ptn, Rarres2, Sema6d, Spp1, Tac1, Tgfb2, Tgfb3, Vegfa, Vegfb, Vegfc, Wnt10a, Wnt4, Wnt5a, Wnt9a*.
**Detoxification enzymes:** *Adh1, Adh5, Adhfe1, Akr1a1, Akr1b1, Akr1c19, Akr1e2, Akr7a2, Aldh16a1, Aldh1a1, Aldh1a2, Aldh1a7, Aldh2, Aldh3a1, Aldh3a2, Aldh3b1, Aldh3b2, Aldh4a1, Aldh5a1, Aldh6a1, Aldh7a1, Chst1, Chst10, Chst11, Chst12, Chst14, Chst2, Chst7, Chst8, Chst9, Eogt, Ephx1, Ephx2, Ephx3, Gal3st4, Gpx1, Gpx3, Gpx4, Gpx6, Gpx7, Gpx8, Gsta1, Gsta4, Gstk1, Gstm1, Gstm2, Gstm4, Gstm5, Gstm6, Gstm7, Gsto1, Gstp1, Gstt1, Gstt2, Gstt3, Gstz1, Hs2st1, Hs3st1, Hs6st1, Hs6st2, Mgst1, Mgst2, Mgst3, Mt1a, Mt1m, Mt2A, Mt3, Ndst1, Ogt, Pomgnt1, Pomgnt2, Sepp1, Sult1a1, Sult2b1, Sult4a1, Tpst1, Tpst2, Txnrd1, Txnrd2*.
**ECM components:** *Agrn, Bgn, Col11a1, Col11a2, Col13a1, Col16a1, Col17a1, Col18a1, Col22a1, Col24a1, Col27a1, Col2a1, Col4a1, Col4a2, Col4a4, Col4a5, Col5a1, Col5a2, Col6a2, Col8a1, Dcn, Ecm1, Efemp2, Emilin1, Fbln1, Fbn1, Fbn2, Fmod, Fn1, Gpc1, Gpc4, Hapln1, Lama1, Lama3, Lama5, Lamb1, Lamb2, Lamc1, Lamc2, Mgp, Prelp, Ptprz1, Sdc1, Sdc2, Sdc3, Sdc4, Smc3, Spock1, Spock2, Spock3, Tgfbr3, Tsku, Vcan*.
**Cell adhesion molecules:** *Alcam, Arvcf, Bcam, Cadm1, Cadm3, Cadm4, Cd47, Cdh1, Cdh11, Cdh13, Cdh15, Cdh18, Cdh2, Cdh22, Cdh24, Cdh26, Cdh4, Cdh6, Cdh8, Cdhr1, Cdhr2, Ceacam16, Celsr1, Celsr2, Celsr3, Cercam, Clstn1, Clstn2, Clstn3, Dchs1, Dsc2, Dscam, Dscaml1, Epcam, F11r, Fat1, Fat3, Fgfrl1, Hepacam2, Itga2b, Itga3, Itgae, Itgav, Itgb1, Itgb5, Itgb8, Jam2, Jam3, Lrfn1, Lrfn3, Lrfn4, Lrrc4c, Lsamp, Mpzl1, Mpzl2, Ncam1, Ncam2, Negr1, Nfasc, Nlgn3, Nrcam, Nrxn1, Nrxn2, Nrxn3, Ntm, Pcdh1, Pcdh10, Pcdh17, Pcdh18, Pcdh19, Pcdh7, Pcdh8, Pcdh9, Pcdhb17, Pcdhb3, Pcdhb5, Pcdhb7, Pcdhga4, Pcdhgb7, Ptprm, Pvrl1, Pvrl2, Pvrl3, Robo2, Sirpa, Thy1, Vcam1*.

*Each gene is expressed in at least 5% of cells in at least one pituitary cell type*.

The subset of dominant genes identified as cell type-specific markers included several notable examples. Among 31 specific marker genes common to all pituitary cells (HPC+FSC) were two development genes, *Pitx1* and *Pitx2*; neuroendocrine genes *Fuz*, and *Hap1*; and a cell adhesion molecule gene *Epcam*. Two small G proteins, *Rab25* and *Rab36*, were also specific markers common to all pituitary cells, as well as *Cldn9*, a member of the claudin family of proteins critical for tight junctions, which control the flow of molecules in the intercellular space. All pituitary cell types, but not E, Le, or EC, also express *Rpl39l*, a gene with high sequence similarity to the ribosomal protein L39, a component of the ribosome 60S subunit (Figure 1C; bold in Table 1).

The 36 genes found to be HPC-specific included the neuroendocrine markers *Resp18, Uchl1, Chgb*, and *Scg2*, which we used to classify these cells (Figure 1B), as well as additional neuroendocrine marker genes, including *Caly, Snap25, Snap91, Stmn3, Syp, Syt4*, and *Syt7*. These cells also specifically expressed *Camk2b*, calcium/calmodulin dependent protein kinase II beta; *Gabrb3*, gamma-aminobutyric acid type A receptor beta3 subunit; *Insm1*, INSM transcriptional repressor 1; *Ptprn2*, protein tyrosine phosphatase receptor type N2v; *Tmem130*, transmembrane protein 130; *Unc80*, unc-80 homolog (Figure 1C; bold in Table 1).

Among FSC-dominant genes, 85 were identified as specific marker genes for these cells. Some of the most well-expressed markers included the commonly accepted FSC marker gene *S100b*, as well as *Fxyd1, Gstm2*, and *Capn6*, which we used to classify FSC (Figure 1B). Several of the development/differentiation genes (*Hey1, Hey2, Heyl, Prop1, Prrx1*, and *Sox9*), endogenous ligand genes (*Cxcl12, Edn3, Gpha2, Mdk, Penk, Rarres2*, and *Wnt5a*), and detoxification enzyme genes (*Aldh1a2, Aldh1a7, Aldh3a1, Gsta4, Gstm2, Gstm6*, and *Gstt3*) were also identified as FSC-specific.

In addition to *Pomc, Crhr1, Avpr1b* (Figure 1B), and *Tbx19*, the well-established marker genes for C, our analysis suggested 15 novel C-specific genes. These included: *Doc2g*, a member of the DOC2 family of proteins that participate in exocytosis; *Chrna1*, encoding neuronal acetylcholine receptor subunit alpha-1; *Lmx1a*, a member of the LIM class of transcription factors; *Fam89a*, family with sequence similarity 89 member A, *Clrn1*, clarin-1, and *Il22*, an endogenous ligand gene (Figure 1C; bold in Table 1).

Of 72 G-dominant genes identified, 24 were G-specific, including *Lhb, Fshb*, and *Gnrhr*, the expected markers for this cell type (Figure 1B). Additional markers included: *Pitx3, Dusp15*, dual specificity phosphatase 15; *Cnga1*, cyclic nucleotide gated channel alpha 1; *Chrna4*, cholinergic receptor nicotinic alpha 4 subunit; *Icam5*, intercellular adhesion molecule 5; *Kcnmb4*, calcium-activated potassium channel subfamily M regulatory beta subunit 4; *Ntrk3*, neurotrophic receptor tyrosine kinase 3; and *Tgfbr3l*, transforming growth factor beta receptor 3 like (Figure 1C; bold in Table 1).

In contrast to C and G, the *Pou1f1*-derived cell types contained fewer dominant and specific genes. Besides *Gh1* and *Ghrhr*, dominantly expressed genes for S included: *Cabp2*, calcium binding protein 2; *Cdkn2a*, cyclin dependent kinase inhibitor 2A; *Nog*, noggin; *Pappa2*, pappalysin 2; and *Dcdc5*, doublcortin domain containing 5 (Figure 1C). Among these only *Pappa2* satisfied our criteria to be a specific marker gene for S. The 13 L-specific genes we identified included not only *Prl, Drd2*, and *Agtr1b*, the well-known marker genes for these cells, but also: *Krt25*, a type-I keratin gene; *Asic4*, an acid sensing ion channel subunit; *Irx6*, iroquois homeobox 6; and *Klk1b3*, kallikrein 1-related peptidase B3 (Figure 1C and Table 1). As expected, T specifically expressed *Tshb*, as well as *Nrg4*, neuregulin 4; *Dpp10*, dipeptidyl peptidase like 10; *Pcsk2*, proprotein convertase subtilisin/kexin type 2; and *Zcchc16*, retrotransposon Gag like 4 (Figure 1C; bold in Table 1).

### Sexually Dimorphic Gene Expression

The tSNE maps shown in Figure 1A do not allow assessment of sexual dimorphism. To address that, we computed a tSNE map containing pituitary cells from both sexes (Figure 2A), demonstrating clear sexual dimorphism in some cell types and not in others. This is clear for L and G, both of which appear as two distinct clusters, whereas for S a subset of female cells appears distinct from a mixed main cluster. FSC also formed two clusters, but both clusters contained a mix of cells from both sexes, indicating that sexual dimorphism was not the cause of subclustering. The tSNE map also suggests very little sexual dimorphism in C, based on uniform mixing of male and female cells in that cluster.

We used differential expression analysis to identify genes dominantly expressed per sex and sex-specific marker genes for pituitary cell types. Figure 2C shows the sex-specific expression of genes in G and L. Table 3 and Figure 2B show the number of genes dominantly expressed by sex in all pituitary cell types. L contained the largest number of sex-dominant genes (288), followed by G (128), S (34), T (14), FSC (10), and C (4). Furthermore, a disproportionately large percentage of such genes were male dominant: C (100%), T (100%), G (77%), L (68%), and S (59%).

**Table 3:**
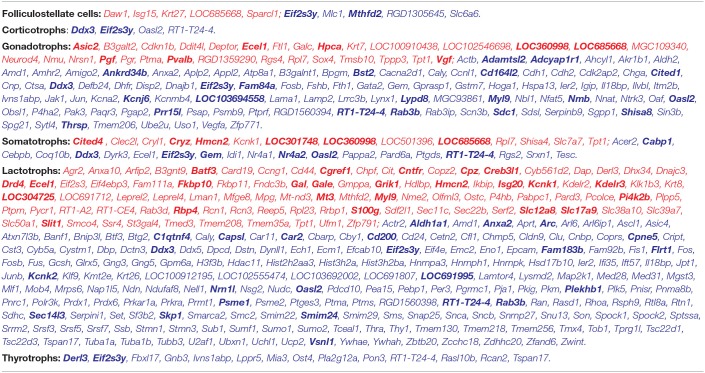
Genes dominantly and specifically expressed in pituitary cells from females and males.

*Significantly upregulated genes (adjusted-P < 0.001) were identified by differential expression tests between cells from females and males of each pituitary cell type. Genes dominantly expressed in the sex of interest for each cell type had at least 20% cells expressing > threshold and at least 3-fold greater mean expression or 30% higher proportion of cells expressing > threshold than the other sex. Red: female specific; blue: male specific; bold indicates sex-specific marker genes, which were sex-dominant genes with <5% of cells expressing > threshold in the opposite sex*.

Table 3 also lists all sex-specific marker genes (bold). In females we identified 24, 8, and 6 markers in L, G, and S, respectively, and none in FSC, C and T, and in males we found 24, 21, 7, 2, 2, and 2 marker genes in L, G, S, T, C, and FSC, respectively. The sexually dimorphic genes identified include genes encoding enzymes, voltage- and ligand-gated plasma membrane channels, endogenous ligands, receptors and intracellular signaling molecules, and transcription factors (Table 3). For example, L from females, but not from males, express *Drd4*, dopamine receptor 4, *Gal*, galanin; *Grik1*, glutamate ionotropic receptor kainate type subunit 1; *Kcnk1*, potassium two pore domain channel subfamily K member 1; and *Pi4k2b*, phosphatidylinositol 4-kinase type 2 beta. In contrast, L from males specifically express *Kcnk2*, potassium two pore domain channel subfamily K member 2; *Nrn1l*, neuritin 1 like; and *Rab3b*, a member of the RAS oncogene family.

Female G cells express *Asic2*, acid sensing ion channel subunit 2; *Pgf*, placental growth factor; *Pvalb*, parvalbumin; and *Vgf*, nerve growth factor inducible. In contrast, G from males express, among others, *Adcyap1r1*, ADCYAP receptor type 1; *Kcnj6*, potassium voltage-gated channel subfamily J member 6; and *Rab3b* (Table 3). As expected, there were low levels of *Fshb* in diestrus females, so that this gene was dominantly expressed in male G. Although they did not meet the strict criteria we used for dominant expression, *Prl* had roughly 2-fold higher expression in female L, as did *Cga* and *Lhb* in female G cells relative to males. The expression of *Eif2s3y* and *Ddx3y*, which are on the Y chromosome, only in male pituitary cells further supports the validity of this analysis (Table 3).

### Genetic Relationship Between FSC and HPC

To study the genetic relationship between FSC and HPC, we focused on two groups of genes suggested by our differential expression analysis: development and neuroendocrine marker genes (Table 2). From these lists of genes, we identified those that were dominantly expressed in at least one pituitary cell type relative to E, Le, and EC. For this subset of genes, we examined in detail five categories of genes: FSC-specific, FSC-dominant, common to FSC and HPC (pituitary-dominant, but not FSC- or HPC-dominant), HPC-dominant for any HPC type, and HPC-specific in any HPC type, as described in Figure 3 legend and Table 1. The remaining genes from Table 2 that were not pituitary cell type-dominant were still expressed in pituitary cells; these were expressed at lower levels than our criteria for dominant expression, were co-expressed in E, Le, or EC and a pituitary cell type, or were dominantly expressed in one of E, Le, or EC.

Figure 3A illustrates expression profiles of development/differentiation genes representative of the following patterns: common to FSC and HPC (*Pitx1*), FSC-dominant (*Egr1*), FSC-specific (*Prop1*), and HPC-specific (C—*Tbx19*; G and T—*Nr5a1*; T, S, L—*Pou1f1*). Figure 3B shows the percentage of cells per type expressing 33 identified pituitary-dominant development genes. In addition to *Pitx1, Pitx2* was also expressed in all pituitary cell types, while *Lhx3* was expressed in L, FSC, G, T, and a low percentage of C. *Sox2* was well expressed in FSC, and in a low percentage of C and G, whereas *Sox9* was exclusively expressed in FSC. *Prrx1*, and *Egr3* were expressed in FSC only; *Gata2* was found not only in G and T, but also in 9% of S; *Neurod1* was expressed in C and G; *Neurod4* in G, T, S, and L; *Pitx3* in G only; and *Sox6* and *Tbx19* in C only. Out of 64 development/differentiation genes expressed in pituitary cells (Table 2), we identified 13 FSC-specific or -dominant genes, 11 HPC-specific or -dominant genes, and 9 genes common to FSC and at least one HPC type (Figure 3B).

Pituitary cells also express numerous genes typically observed in neuroendocrine cells. Among 99 neuroendocrine marker genes expressed in at least 5% of cells in at least one pituitary cell type (Table 2), 70 were dominantly expressed in one or more pituitary cell types (Table S1). Figure 4A shows examples of such genes dominantly expressed in FSC and some HPC (*Rtn1*), HPC-dominant (*Bex2*), expressed in some HPC types only (*Scg2, Chgb, Caly*, and *Syt4*), in FSC only (*Ndrg2*), and in C only (*Doc2g*). Figure 4B shows that most these genes were dominantly or specifically expressed by HPC or co-expressed in FSC and HPC. Many of these genes were widely expressed in HPC; 28 were expressed in at least 80% of one or more HPC types. Among this gene group, *Fuz, Gnas, Nptn*, and *Rtn1* were expressed in all pituitary cell types. *Snap25, Snap91, Stmn3, Syt4*, and *Syt7* were well-expressed in all HPC types and were identified as specific markers for this population of pituitary cells (Table 1). In contrast to *Chgb*, the expression of *Chga* was relatively low in C, which uniquely expressed *Doc2g*, a marker gene for this cell type. *Ndrg2* and *Tagln* were expressed dominantly and specifically in FSC, respectively. In total, only 9 genes were FSC-specific or -dominant, 47 were HPC-specific or -dominant, and 14 were dominantly co-expressed by both FSC and HPC types. The enrichment of neuroendocrine genes related to exocytosis in HPC is consistent with the physiological role of hormone secretion in these cells.

### Functional Relationship Between FSC and HPC

To further study the relationship between FSC and HPC, we similarly analyzed four additional groups of genes: endogenous ligands, detoxification enzymes, ECM proteins, and cell adhesion proteins. Figure 5A shows expression profiles of some endogenous ligand genes: *Il33* and *Edn3* in FSC, *Ptn* in FSC and L, *Dlk1* in S, T, L, and C, and *Cxcl14, C1qtnf4, Anxa5*, and *Lgals1* in most pituitary cell types. Among 94 endogenous ligand genes expressed in at least 5% of cells in at least one pituitary cell type (Table 2), a total of 44 endogenous ligand genes were dominantly expressed in at least one pituitary cell type (Table S2). For the 33 most highly expressed genes, Figure 5B shows the percentage of each cell type expressing genes from this group. Fifteen genes were FSC-specific or -dominant, including *Anxa1, Cxcl12, Igfbp2, Mdk*, and *Penk*; 18 genes were HPC-specific or -dominant, including *C1qtnf4* and *Fgf9* in all HPC, *Inha*, and *Vegfa* dominant in G, and *Bmp15* and *Nmu* in T; and 11 genes were common among FSC and one or more HPC type, including *Anxa7, Ccl27, Copa*, and *Cntn1*.

Expression profiles for select genes encoding detoxification enzymes are shown in Figure 6A: *Gstm1* and *Gpx8* dominantly in FSC, *Gpx3* dominantly in all HPC, *Mgst1* and *Mt3* highly expressed in FSC with weak expression in HPC, *Aldh1a1* expressed in FSC and L (44), *Aldh2* in FSC and G, and *Adh1* in C only. Most well-expressed genes were found in FSC; among HPC, G also express well genes from this group (Figure 6B). Among 79 detoxification enzyme genes expressed in pituitary cells (Table 2), 20 were FSC-specific or -dominant, 6 were common for FSC and HPC, and only 7 genes were HPC-specific or -dominant.

Figure 7A shows four examples of expression pattern of ECM protein genes: *Prelp* and *Col6a2* in FSC, and *Sdc2* and *Gpc4* in all pituitary cell types. Figure 7B indicates a comparable number of pituitary dominant genes distributed between FSC and HPC. Among 53 ECM genes expressed in pituitary cells (Table 2), only 5 were FSC dominant, 8 HPC-dominant, and 3 common to pituitary cells. Only a few genes were expressed by all cell types: *Sdc2, Spock2, Col11a1*, and *Gpc4*.

Figure 8A shows expression profiles of cell adhesion molecule transcripts. *Epcam* and *Cadm1* are expressed in all pituitary cell types, *Nrxn1* in C, G, FSC, and T; *Nrcam* is expressed dominantly in FSC, *Thy1* is expressed in T, S, and L, whereas *Cdh1* was expressed in some FSC and G. Figure 8B shows all 29 cell adhesion genes that were dominantly expressed in at least one pituitary cell type. These genes were distributed between FSC and HPC, with a slight bias toward HPC dominant expression. Among 87 cell adhesion genes expressed in pituitary cells (Table 2), 7 were FSC dominant, 15 HPC-dominant, and 7 common to pituitary cells.

### Identification of Proliferating Cells

Figure 1A indicates the existence of a small cluster of cells expressing cell cycle genes in both sexes (circled). We used an iterative method (Materials and Methods) to identify a set of 39 cell cycle marker genes with Gene Ontology annotation “mitotic cell cycle” (GO:0000278) upregulated in these cells, pooling both sexes together (Figure 9; Table S3). These marker genes were used to compute a cell cycle score for each cell, defined as the number of markers expressed above threshold in that cell (Figure 9A). Cells were classified as proliferating if at least 14 of these cell cycle markers were expressed > threshold (dark gray, 108 cells; Figure 9B). This low percentage of proliferating cells is consistent with previous reports (25), as is the fact that the majority of these cells were *Pou1f1* positive (27 S cells and 26 L cells). The remaining proliferative cells were mostly FSC (39 cells), Le (9 cells), EC (5 cells), and C (2 cells). No cycling cells were observed in E, G, and T. Expression of the top 15 expressed cell cycle markers per cell type is shown in Figure 9C.

### Validation of scRNA-seq Analysis

**Figure 10:**
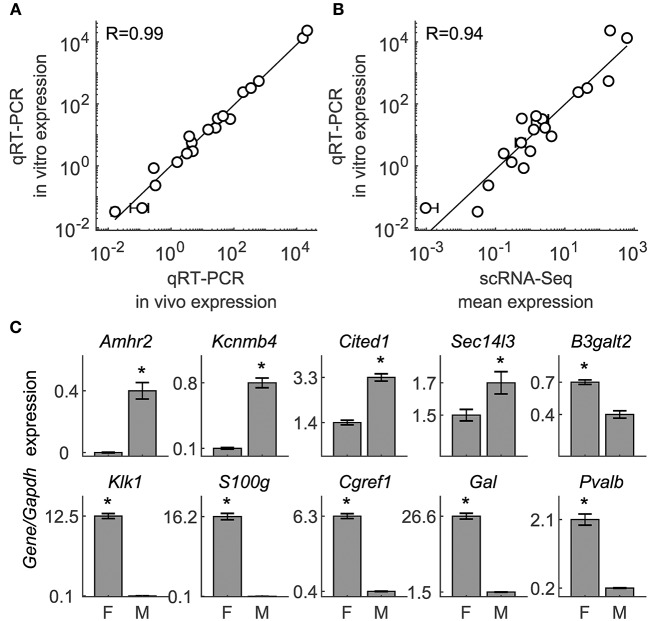
Validation of expression for selected genes by qRT-PCR. **(A)** Measurement of expression by qRT-PCR of 19 genes in dispersed cells is highly correlated with that measured in anterior pituitary tissue from diestrus female rats. **(B)** Correlation of measurements of mean expression in dispersed pituitary cells by scRNA-seq and qRT-PCR. Genes represented: *Caly, Cga, Fshb, Gata2, Gfap, Gh1, Lhb, Lhx3, Lhx4, Pitx3, Pomc, Prl, S100b, Sez6l2, Snap25, Sox2, Stmn3, Tmem130*, and *Tshb*; expression values are shown in Table S4. **(C)** Validation of 10 genes identified as sexually dimorphic in scRNA-seq by qRT-PCR expression measurement in dispersed cells (compare with Figure 2C). Asterisks indicate statistical significance at the *p* = 0.05 level by two-sample *t*-test with unequal variances, *n* = 6 samples per sex.

To examine the validity of our scRNA-seq results, we performed two types of experiments using qRT-PCR analysis. First, we compared the expression of selected genes estimated by scRNA-seq to expression measured by qRT-PCR in anterior pituitary tissue and dispersed cells from postpubertal female rat anterior pituitaries. We selected 19 genes, including hormones and cell type marker genes, HPC-specific markers, and development/differentiation genes (Table S4). We found a tight correlation between tissue and dispersed cell expression of these genes in qRT-PCR measurements (Figure 10A), as well as between qRT-PCR and scRNA-seq data for dispersed cells (Figure 10B). Consistent with scRNA-seq results, our qRT-PCR analysis revealed very low expression of *Gfap*, a marker gene for posterior pituitary cells (45), confirming that our anterior pituitary cells were not contaminated with posterior pituitary cells (Table S4).

Second, we compared the sex-specific expression of selected genes estimated by scRNA-seq to expression measured by qRT-PCR in freshly dispersed cells from postpubertal male and female rats. To do this, we selected 10 of the 40 genes shown in Figure 2C to be expressed in a sex-specific manner. Consistent with scRNA-seq data, qRT-PCR analysis revealed significantly higher expression of *B3galt2, Cref1, Gal, Klik1b3, Pvalb*, and *S100g* in cells from females, whereas *Amhr2, Cited1, Kcnmb4*, and *Sec14l3* where significantly higher expressed in cells from males (Figure 10C).

**Figure 11:**
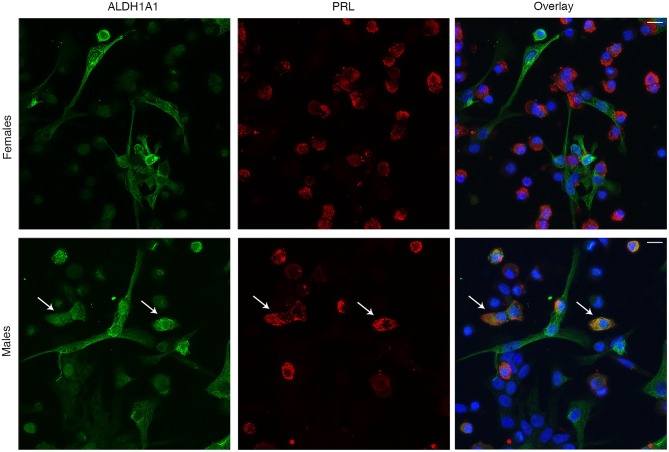
Immunofluorescence analysis of sexual dimorphism in ALDH1A1 expression in anterior pituitary cells. Expression of ALDH1A1 (green, left), PRL (red, center), and their overlay (right) in females (top panels) and males (bottom panels). Cell nuclei are stained with DAPI (blue). Arrows indicate example cells that co-express both ALDH1A1 and PRL. Scale bars (applies to all images), 10 μm.

We also used immunocytochemistry of dispersed pituitary cells from female and male rats to examine expression of selected proteins and compare it with scRNA-seq data. First, we examined the expression pattern of ALDH1A1, which gene was found to be expressed in L from males but not from females, and in FSC from both sexes. In parallel to scRNA-seq data, among 293 PRL-positive cells from females we found only 8 of them (3%) to be positive for ALDH1A1 (Figure 11, top panel). However, in males, 94 cells out of 212 PRL-positive cells (44%) co-expressed ALDH1A1 (bottom panel). Figure 11 also indicates the presence of ALDHA1 staining in PRL-negative cells. The shape of these cells is consistent with the expression of ALDH1A1 in FSC. Double immunostaining with S100B and ALDH1A1 confirmed this hypothesis (Figure S3). In contrast to L, however, we did not observe a difference in respect to sex; in both sexes around half of S100B-positive cells were also positive for ALDH1A1 (54% for females and 48% for males).

**Figure 12:**
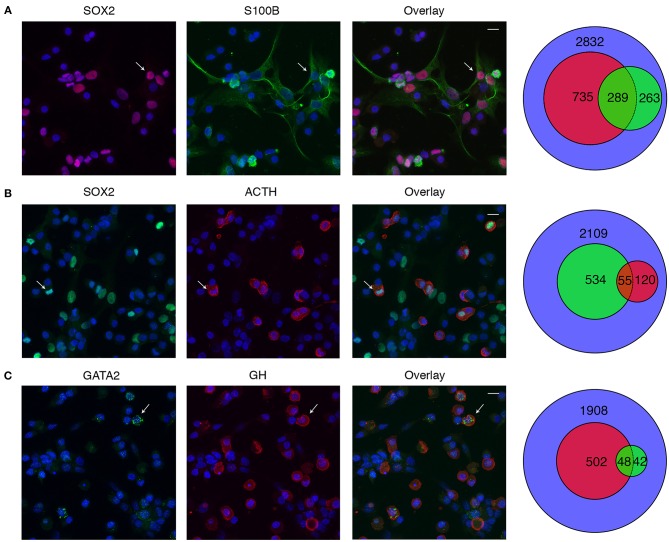
Immunofluorescence analysis of SOX2 and GATA2 expression in anterior pituitary cells. **(A)** SOX2 (red, column one), S100B (green, column two), and their overlay (column three). Cell nuclei are stained with DAPI (blue). The Venn diagram (column four) shows the total numbers of nuclei (blue), SOX2+ (red), S100B+ (green), and dual labeled cells counted. **(B)** SOX2 (green, column one), ACTH (red, column two), and their overlay (column three). The Venn diagram shows counts of SOX2+ (green), ACTH+ (red), and dual labeled cells counted. **(C)** GATA2 (green, column one), GH (red, column two), and their overlay (column three). The Venn diagram shows counts of GATA2+ (green), GH+ (red), and dual labeled cells counted. Arrows indicate an example cell that co-expresses both proteins. Scale bars (applies to all images), 10 μm.

We also evaluated the expression of SOX2 and GATA2 proteins to test unexpected expression of *Sox2* in C and *Gata2* in somatotrophs observed in scRNA-seq analysis. Anterior pituitary cells were incubated with rabbit anti-SOX2 antibody, followed by incubation with either mouse anti-S100B antibody or guinea pig anti-ACTH antibody. Figures 12A,B show representative data of SOX2-positive cells (first column), S100B/ACTH-positive cells (second column) and their overlay (third column). The Venn diagram, shown in fourth column, quantitatively summarizes these data. Of 2,832 cells analyzed, 1,024 cells were SOX2-positive, while 552 cells were S100B positive. Of the S100B-positive cells, 289 cells were also positive for SOX2 (Figure 12A). Additional experiments revealed that out of 2,109 cells, 589 cells were SOX2-positive and 175 cells were ACTH-positive. Of the ACTH-positive cells, 55 cells were also SOX2-positive (Figure 12B). To evaluate GATA2 expression in S, dispersed anterior pituitary cells were incubated with rabbit anti-GATA2 antibody, followed by incubation with guinea pig anti-GH antibody. Out of 1,908 cells, GATA2 immunoreactivity was detected in 90 cells whereas 550 cells were GH-positive. Co-expression of both proteins was observed only in 48 cells (~8%) of the GH-positive cells, consistent with the 9% of *Gata2*-positive S in scRNA-seq.

*S100b* is generally accepted as a marker gene for FSC in anterior pituitary (13). Our scRNA-seq analysis revealed that FSC, classified using multiple marker genes (Materials and Methods), represented ~34% of the cells in our cell preparation, whereas *S100b*-positive cells represented 27% of cells. To validate this aspect of our cell preparation, we evaluated the percentage of cells expressing S100B proteins in our double immunocytochemical studies with ALDH1A1/S100B and SOX2/S100B, and single S100B immunostaining done with anterior pituitary cells from males and females. In six independent experiments, the percentage of S100B-positive cells varied between 20 and 33%, with mean values of 25.6 ± 1.9%.

There have been reports of bihormonal cells in the anterior pituitary, including lacto-somatotrophs that produce both GH and PRL (46). We did not find cells expressing both *Gh1* and *Prl* above threshold, so we performed additional immunocytochemistry experiments to clarify this issue (Figure S4). Overnight-cultured anterior pituitary cells were incubated with rabbit anti-GH antibody, followed by incubation with guinea pig anti-PRL antibody. We used two dilutions for both antibodies: 1:250 and 1:500 for GH; 1:1,000 and 1:2,000 for PRL. Out of 5,238 analyzed cells, 2,768 cells were positive either for GH or PRL, but we did not observe co-expression of these proteins in any of the cells. Representative data are shown in Figure S4. These data do not exclude the existence of lacto-somatotrophs in intact anterior pituitary tissue but confirmed the validity of our scRNA-seq analysis.

## Discussion

Dissociation of cells from a tissue followed by scRNA-seq of freshly dispersed cells provides the possibility to group the cells based on their genetic profiles and frequently leads to discovery of new subpopulations or population of cells for this tissue (47, 48). scRNA-seq also provides an excellent tool to identify typical genes for subpopulations within a tissue or organ, which is critical for cell type definition and provides a useful tool for cell type-specific labeling and manipulation (49). Here we present scRNA-seq observations of adult male and female rat anterior pituitary cells along with an analysis of cell type- and sex-dependent transcriptome profiles.

This method also has some limitations that we and others have encountered. In scRNA-seq technologies such as the 10X Genomics Chromium system used here, a polyT primer is used to target the polyA region of fully transcribed mRNAs. As such, reads will map to the distal end of the 3′UTR. We found many genes, expected *a priori* to be well-expressed in pituitary cells, for which reads mapped downstream of the 3′UTR position annotated in the NCBI Rnor6 reference genome, as has been reported in other recent studies (25, 28, 29). In those studies, one or more 3′UTR annotations were manually extended using the full-length libraries constructed using bulk RNA-seq as a guide, with extensions ranging from 452 to 10.8 kb. Here, we opted to extend all 3′UTRs by the lesser of 4 kb and the distance to the nearest genomic feature (including all non-coding gene types). This resulted in recovered counts for several genes of pituitary interest, including *Prop1, Egr1, Isl1, Gnrhr, Smad4, Gnaq*, and *Fgf9*, along with several hundred others. This automated method of extension could be improved by a more systematic comparison to full length transcripts, and such efforts could be eventually be used to improve the reference genome annotations.

Our tSNE mapping of anterior pituitary cells was consistent with classic knowledge about the presence of five populations of HPC, as well as FSC and EC, which are critical to pituitary function (18). We also observed a very small number of pericytes but did not analyze them here. We introduced a two-step differential expression analysis to identify dominant and cell-type specific (marker) genes for anterior pituitary cell populations. This analysis shows that two endogenous pituitary cell populations, FSC and HPC, express several common marker genes, including *Pitx1, Pitx2*, and some neuroendocrine markers, but each population is also characterized by specific marker genes. Consistent with the literature, our analysis confirms that *Resp18* (50)*, Uchl1* (51), and *Snap25* (52) are good cell markers for all HPC. It has been reported that *Chgb* (53), *Ptprn2* (54), *Scg2* (55), and *Syp* (56) are expressed in some HPC types, but here we show that these genes are marker genes for all HPC. In addition, we identified 30 novel specific markers common to all HPC types.

Gonadotrophs expressed the expected marker genes *Lhb, Fshb*, and *Gnrhr* (57), as well as *Spp1* (58), *Nr5a1* (59), and *Amhr2* (60). We also identified 18 novel markers and numerous dominant genes for G. In addition to the well-established markers for C, *Pomc, Crhr1, Avpr1b*, and *Tbx19* (61), here we provide evidence for 15 novel markers for these cells. The marker genes for L are *Prl, Agtr1b, Drd2*, and *Gal* (62), with 9 novel genes suggested here. The commonly accepted markers for S are *Ghrh1, Gh1*, and *Sstr2* (63). Our analysis identified the first two genes in S only, whereas *Sstr2* was co-expressed by S and T, and we also found that *Pappa2* is a marker gene for S. Finally, our data confirmed that *Tshb* is a marker gene for T. We were unable to detect *Drd2* expression in T, arguing against a hypothesis that dopamine plays a role in control of these cells' function (64), but we identified five novel markers for these cells. The discovery of these novel marker genes for HPC types not only provides tools for their identification, but also points to further studies to evaluate the role of these genes in cell type-specific functions.

A recent study reported scRNA-seq data of male pituitary cells (25). In contrast to our study, their experiments were done with mouse pituitary cells and included the intermediate and posterior lobes, in addition to the anterior lobe of pituitary. They were also able to identify five HPC types expressing known marker genes for these cells and to list additional genes typical for these cells. There is overlap of our dominant genes with the markers identified in their study; for example, *Tgfbr3l, Spp1, Foxp2* for G, *Dio2* for T, *Pappa2* and *Cabp* for S, and *Hepacam2* for L. Some genes listed in their study as novel marker genes for HPC types were also detected in our study, but they do not satisfy our stricter criteria for listing as dominant and specific marker genes. We should not exclude that species differences could also contribute to differences in novel marker genes for HPC types. We found a similar set of cells expressing cell cycle marker genes that predominantly comprised *Pou1f1*-expressing cells, with a similarly small fraction of cells (~1.5% of cells in our study and ~1.25% in their study), suggesting low rates of proliferation in anterior pituitary gland at this life stage. All the markers identified for proliferating cells in their study were also found in our proliferating cells, and all but two (*Hmmr* and *Cdca3*) were identified as markers using our iterative method (Methods and Materials). We present here a detailed description of FSC genes and a comparison with HPC, which was not addressed in their study.

Our data clearly show that sex-specificity in expression of genes accounts for G and L subclusters observed in our sex-combined tSNE map (Figure 2A). We also observed significant differences in expression of genes in S from males and females, but very little sexual dimorphism in C and FSC. Previously, we reported larger differences in expression of those genes in L, G and S, but not in C during development (27). Here we report elevated expression of *Prl* (in L), *Cga* (G), and *Lhb* (G) in females, and elevation of *Fshb* (G) in males, although only *Fshb* met our criteria for sex-dominant expression. In contrast, no sex-specificity in *Pomc* (in C), *Cga* and *Tshb* (T), and *Gnrhr* (G) was observed. Our analysis identified many genes in addition to these hormone genes that may underlie the sexual dimorphism observed in some cell types. The list of 288 sex-dominant genes in L is impressive and provides a solid base for further work on this topic. In G we identified 128 genes expressed in a sex-specific manner, and others have observed an impressive diversity in expression of specific groups of genes in purified G from males and females during development and in cycling females (24). In agreement with that study, we found that *Asic2* was dominantly expressed in our diestrus female G cells, while *Adcyap1r1, Gata2*, and *Jun* are male-dominantly expressed.

In a recent study, the sex-specific changes in expression of 48 pituitary genes was detected across postnatal mouse development (65). In postpubertal rats, we detected the expression of 34 of these genes in more than 5% of cells, mostly in a sex-non-specific manner, indicating a transient nature of sex-specific expression during puberty. However, the sex-specific expression was preserved for some genes postpubertally. Among them, only two of these genes were listed as sex-specific using our criteria stated in Table 3: *Pgr*, expressed dominantly in G from females, and *Fshb*, expressed dominantly in G from males. With a less restrictive criteria (at least 2-fold higher proportion of cells expressing genes than the other sex), several other genes were expressed in a sex-specific manner; G from postupubertal females expressed more *Bdnf* and *Kiss1*, whereas postpubertal males expressed more *Begain* (L), *Map2k5* (L) and *Pcsk1* (T). We also detected sex-specific expression in the opposite sex from that reported previously for a few genes: *Gnpda2* (L), *Reep3* (T), *Tyw*3 (L), and Ntrk2 (G).

One of the conclusions of work with male mouse scRNA-seq was that individual HPC types form subclusters (25). In general, among cells of a given sex, this could be consistent with literature data about bihormonal cells found in anterior pituitary, lacto-somatotroph (46) and/or gonado-somatotroph (19) for example. We did not find evidence of these bihormonal subtypes of HPC based on scRNA-seq analysis or immunocytochemistry in our cells, so we cannot comment on their transcriptional profiles. We do not exclude the possibility that such bihormonal cells were selected against during cell dispersion. Alternatively, pituitary cell subtypes observed in scRNA-seq could reflect cells with specializations in function or separate positions in the gland. Further work should be done to better assess the presence and characteristics of pituitary cell subtypes.

FSC have been reported to represent 4–20% of pituitary cells (15, 66, 67); they are chromophobes (12), and *S100b* is generally accepted as their marker gene (13). Our scRNA-seq and immunocytochemical analyses revealed that FSC represented a larger percentage of all anterior pituitary cells in our preparation (scRNA-seq, ~34% of cells classified as FSC, with ~27% of cells expressed *S100b* greater than threshold; immunocytochemistry, between 20 and 33% S100B-positive cells, with mean values of 25.6 ± 1.9%). This might reflect differences in animal age and cell dispersion protocols between studies. Possible contamination with posterior pituitary cells, which also express S100B (68) was excluded by the finding that *Gfap*, a posterior pituitary-specific gene (45), was essentially undetected by scRNA-seq or qRT-PCR. It has also been suggested that pituitary chromophobe cells are a heterogeneous population composed of typical FSC and other cell types classified as follicular, marginal, mesenchymal, and immune cells, and that some of these cells could be candidates for progenitor cells (69). Our transcriptome analysis does not shed light on such a morphological distinction, but the tSNE map suggests two genetically diverse FSC subtypes present in both sexes. Future work based on this dataset is expected to reveal novel information regarding FSC heterogeneity.

Our data may, however, suggest links between FSC and progenitor cells. In a previous report of scRNA-seq in male mice (25), a cluster of pituitary progenitor cells was defined based on *Sox2* expression alone. In our dataset, *Sox2* was co-expressed with *S100b*, the accepted marker of FSC, in both scRNA-seq (Figure 3) and immunocytochemistry (Figure 12) as has been reported by others (14). The expression of *S100b* was not examined in their study. We note that most of the genes they identified as significantly upregulated in their *Sox2* cluster, including *Lcn2, Aldh3a1, Aldh1a2, Rbpms, Cdh26, Cpxm2*, and *Sox2*, were dominantly or specifically expressed in our FSCs, and that *Aqp4* was found in 15% of FSC but not in other cell types. This suggests that these may reflect similar populations of cells.

Development and differentiation of HPC have been extensively investigated (70–72). It has also been repeatedly suggested that FSC may serve as pituitary progenitor cells (14, 15) and that FSC and HPC are derived from the same pool of cells (16). Our analysis identified several genes expressed among both FSC and HPC from a group of 64 genes known to contribute to pituitary development and differentiation. Furthermore, we found numerous neuroendocrine marker genes to be expressed in both populations of pituitary cells. The co-expression of development genes, such as *Pitx1, Pitx2*, and *Lhx3*, as well as several neuroendocrine markers in both populations of pituitary cells suggests that HPC and FSC may be viewed as sister cells, i.e., cells of a common origin, or that a subset of FSC are pituitary stem cells, as discussed in the literature (69).

We also demonstrated some novel expression patterns of these genes. For example, *Sox2*-positive cells are believed to represent 3–5% of cells in the anterior lobe in adult animals, usually co-expressing *Sox9* and considered to be the stem cells of anterior pituitary (5). We found that 69% and 53% of FSC expressed *Sox2* and *Sox9*, respectively, and that *Sox2* was also expressed in a fraction of C and T. This finding was confirmed by immunocytochemistry (Figure 12). Others have also reported that *Sox2*/*Sox10* expressing cells account for the generation of *S100b*-positive cells (73), but *Sox10* was not detected in our dataset. *Gata2* is well-established to be critical in the development of T (together with *Pou1f1*) and G (without *Pou1f1*) (74). Consistent with this, we observed the expression of *Gata2* in most G and T cells. However, we observed that about 9% of S also express this gene, as well as that 8% of GH positive cells co-express GATA2 protein, indicating heterogeneity of this cell type and a potential role of *Gata2* in its differentiation/function. It has been indicated that *Six3* and *Six6* regulate G-specific gene expression (75). We observed the expression of *Six6* in FSC and all HPC types (predominantly in L and C), whereas *Six3* was expressed in FSC and L only. These findings indicate that in the rat, *Six3* is not essential for postnatal expression of G-specific genes and that both genes may have functions in other cell types, including FSC.

Pituitary cells expressed genes encoding endogenous ligands, which predominantly serve as autocrine/paracrine factors for G-protein coupled receptors, tyrosine kinase receptors, ligand-gated receptor channels, and cytokine/chemokine receptors. Numerous endogenous ligands have been suggested to be secreted by pituitary cells and to act in a paracrine manner (76). Among them we found only a few genes expressed in the indicated cell type: *Pdyn* (G), *Tac1* (T, S), *Vegfb* and *Vegfc* (all pituitary cells), *Inha* and *Inhbb* for potential to produce inhibin B and activin B (G), and *Anxa1* (FSC). Our study confirms that the following genes were expressed in pituitary cells, but the cell types expressing them differed from those listed by Denef (76): *Gal, Nmb, Nmu, Nppc, Penk, Agt, Calca, Edn3, Cartpt, Tgfb3, Inhba, Mif, Vegfa, Angpt1*, and *Fst*. Expression patterns of the following genes encoding endogenous ligands could not be assessed as they were expressed in <5% of cells in all cell types: *Vip, Adcyap1, Grp, Crh, Ucn, Ucn2, Npy, Nts, Adm2, Edn1, Edn2, Avp, Oxt, Ghrl, Hcrt, Fgf2, Tgfa, Lif, Il1a, Il6*, and *Lep*. However, in addition to 44 genes for endogenous ligands confirmed to be dominantly expressed in at least one pituitary cell type, we identified 50 other such genes expressed above the 5% threshold. These findings will guide further work on physiological relevance of these novel genes and crosstalk among FSC and HPC.

Complex enzyme systems protect organisms from a wide array of xenobiotics, from food components to environmental toxins to pharmaceuticals, with liver playing a major role in these processes (77). Other tissues also have mechanisms for protection. The roles of these enzymes in pituitary functions have not been investigated systematically. Earlier work has proposed scavenger functions for FSC, focusing on their role in clusterin removal and metabolism of ammonia (13). Our list of at least 79 detoxification enzyme genes expressed in pituitary cells clearly indicates that this process is complex, that FSC play a major role in this function, and that HPC also contribute to this function. For example, here we identify for the first time that pituitary cells express 13 of 19 known aldehyde dehydrogenase genes, and six of them are dominantly expressed in FSC: *Aldh1a1, Aldh1a2, Aldh1a7, Aldh2, Aldh3a1*, and *Aldh3b1*. These enzymes catalyze the oxidation of aldehydes to carboxylic acids. They are involved in the metabolism of alcohols formed endogenously and xenobiotics that contain alcohol groups, and they further metabolize alcohol metabolites of xenobiotics. Here we also show that male but not female L express the *Aldh1a1* gene and ALDH1A1 protein, which has androgen binding activity (78). Carbohydrate sulfotransferase genes are also expressed in pituitary cells; the enzymes transfer sulfate to carbohydrate groups in glycoproteins and glycolipids. Two of these genes are specific for FSC, others are expressed in HPC as well. Our search revealed that only one of these enzymes have been previously identified in pituitary (79).

The ECM provides physical scaffolding and biochemical support to the surrounding cells, and is composed of proteins and polysaccharides secreted by cells (80). Here we identified a list of 53 genes encoding ECM proteins that were expressed in pituitary cells. Among them, collagens are ECM proteins encoded by over 40 genes. The expression of collagen 1–4 in the pituitary gland has been reported (81). Here we provide novel evidence for expression of 18 members of this family of genes; 11 of these genes were expressed in FSC, whereas *Col2a1, Col8a1, Col11a2, Col16a1, Col17a1*, and *Col24a1* are expressed in HPC only. The expression and role of laminin in gap junction formation in FSC has also been reported earlier (82), as well as that laminin and collagen modulate expression of fibromodulin in FSC (83). There are 12 members of the laminin gene family, and here we show that seven of them are expressed in anterior pituitary cells. Both FSC and HPC express these genes in a cell-type specific manner. FSC have also been suggested to express small leucine-rich proteoglycans (35), and we confirm here expression of *Bgn, Fmod*, and *Prelp* in FSC. Together, these findings suggest the cell-type specificity in the formation of the extracellular space structural proteins in anterior pituitary gland.

Cell adhesion molecule genes encode proteins allowing cells to bind to other cells or the ECM. They typically comprise an intracellular domain that interacts with the cytoskeleton, a transmembrane domain, and an extracellular domain that interacts with another cell adhesion molecule or ECM component (84). This group contains six subgroups of genes with over 300 members. Among them, cadherins are the largest group. Cadherins are calcium-dependent adhesion molecules whose role in the formation of S, L, and C tumors was studied previously (85, 86), but not their expression pattern in anterior pituitary cells. Here we show that both type I classical and type II classical cadherin genes are expressed in pituitary cells. Out of 18 genes of these two families, we identified 12 genes in anterior pituitary; six genes are expressed only in FSC, four for HPC, and 2 are expressed in both FSC and HPC. Protocadherins also constitute a large family of genes encoding proteins composed of up to seven cadherin-like motifs that are distinct from those present in classical cadherins. Many of these proteins are highly expressed in the nervous system and are frequently localized to synapses (87). Here we show that protocadherin genes are also expressed in anterior pituitary. We identified 14 of these genes expressed in a cell type-specific manner. Integrins are cell membrane heterodimeric receptors that mediate cell-to-cell and cell-to-matrix adhesion. The expression and roles of integrins in the pituitary gland have been studied previously (88, 89). Here we show that seven genes from this family are also expressed in anterior pituitary; all of them were identified in FSC and *Itga3, Itgab1*, and *Itgae* in all HPC types as well. Finally, the expression of claudins 2, 4, and 5 in FSC and EC (90) has been reported, but we have not found evidence that these genes were expressed in more than 5% of anterior pituitary cell types.

In conclusion, we used 10X Genomics Chromium Single Cell System-generated data from freshly dispersed cells and detailed differential expression analyses to better understand the complexity of anterior pituitary cells. Our transcriptome survey, combined with selective immunocytochemistry and qRT-PCR analysis, is consistent with the existence of five HPC types, critical for anterior pituitary gland function as a central endocrine regulator of numerous physiological functions including reproduction, growth, metabolism, and stress responses. Among HPC, the expression of cell type-specific genes is most noticeable within corticotroph and gonadotroph lineages, whereas sex specificity in gene expression is most prominent within the lactotroph, gonadotroph, and somatotroph lineages. Our analysis also points to impressive transcriptome diversity of FSC, as well as the expression of pituitary stem/progenitor marker genes in FSC and HPC. The expression pattern of two group of genes, which we named development/differentiation and neuroendocrine genes, unquestionably demonstrates that FSC and HPC are sister cells. Identification of cell type-dominant and -specific genes also points to complex interactions between HPC and FSC in the production of endogenous ligands and detoxification enzymes, organization of ECM, and expression of cell adhesion molecules. Further analysis of other gene families, including those encoding receptors, channels/transporters, intracellular signaling molecules, and transcription factors will help refine these relationships. This and similar studies will be crucial guides for future experimental and clinical investigations into pituitary functions and related disorders.

## Data Availability

The datasets generated for this study have been deposited in NCBI's Gene Expression Omnibus (30) and are accessible through GEO Series accession number GSE132224 (https://www.ncbi.nlm.nih.gov/geo/query/acc.cgi?acc=GSE132224).

## Ethics Statement

The animal study was reviewed and approved by the NICHD Animal Care and Use Committee (16-041).

## Author Contributions

PF, SC, and SS: conceptualization. PF: software. PF and JI: formal analysis. PF, KS, RP, JI, TL, MR, and SS: investigation. SC and SS: resources. PF and JI: data curation. PF and SS: writing—original draft. PF, SC, AS, and SS: writing—review and editing. PF, KS, and SS: visualization. SC, AS, and SS: supervision.

### Conflict of Interest Statement

The authors declare that the research was conducted in the absence of any commercial or financial relationships that could be construed as a potential conflict of interest.
