# Supplementary material for: Cell Type- and Sex-Dependent Transcriptome Profiles of Rat Anterior Pituitary Cells
Source: Front Endocrinol (Lausanne). 2019 Sep 18;10:623. doi: 10.3389/fendo.2019.00623 (PMC6760010; doi:10.3389/fendo.2019.00623)
Supplement: Supplementary file 1 [file Data_Sheet_1.docx]

Supplementary Material

## Supplementary Figures


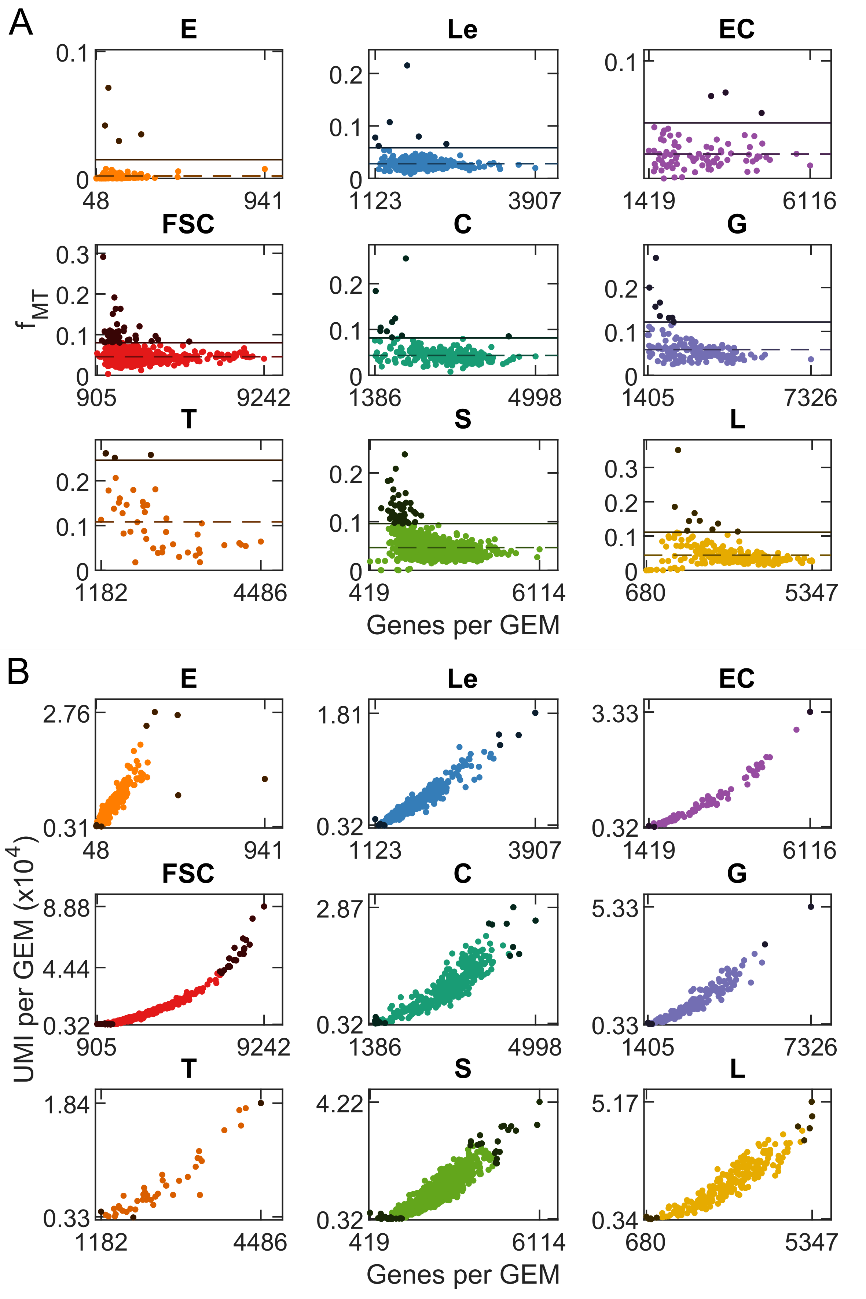


**Figure S1. Cell type-dependent identification of cells with high mitochondrial transcript fraction and gene and UMI count outlier cells.** *A*, For each cell type, the mean (dashed line) and standard deviation of the fraction of mitochondrial transcripts per cell (f_MT_) was computed. Cells were excluded (dark points) if they had greater than mean + 2 SD f_MT_ (solid line). Remaining cells are indicated with colored points. *B*, For each cell type, cells were excluded (dark symbols) if they were in the lowest or highest 1.5% of the distributions of genes per GEM or total UMI counts per GEM. Remaining cells are indicated with colored points. Identification of f_MT_ and gene/UMI outliers was done independently for each sex; shown here are female cells.


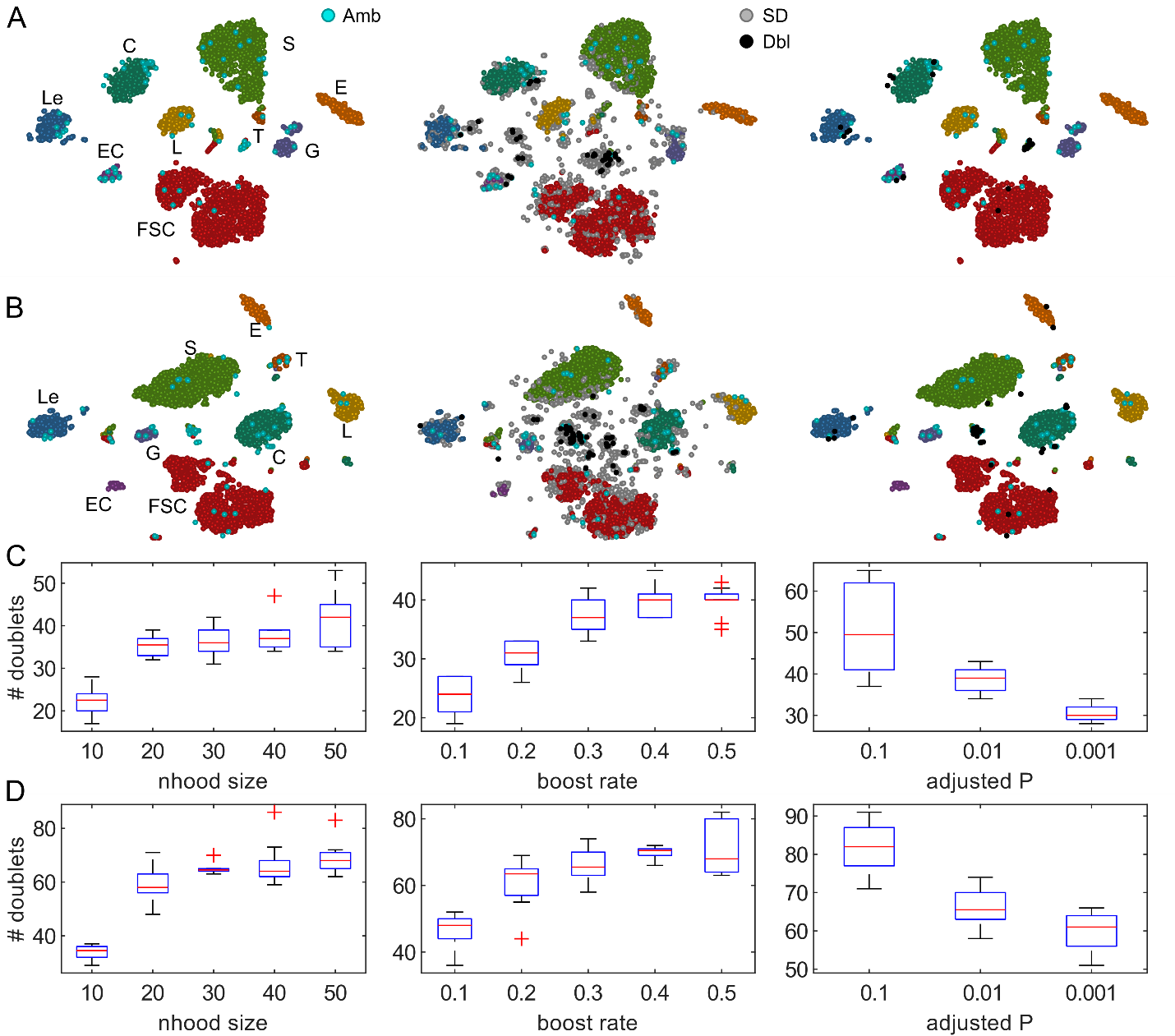


**Figure S2. Computational detection of doublet GEMs.** *A*-*B*, tSNE maps illustrating the doublet detection algorithm for female (*A*) and male (*B*) cells with default parameters kNN = 30, boost rate = 30%, and adjusted-P value cutoff = 0.01. *Left*, cell types identified by marker-based classification and ambiguous cells that satisfied more than one cell type definition (Amb, cyan). *Center*, the synthetic doublet (SD, gray) boosted cell population. Heterotypic doublet cells tend to form clusters separate from true cell clusters, and homotypic doublets cluster homogeneously with parent cells. Cells are identified as doublets (Dbl, black) by k-nearest neighbor classification if sufficiently many of their kNN=30 nearest neighbors are synthetic doublets (see Materials and Methods). *Right*, tSNE map without synthetic doublets showing identified doublet cells. *C-D*, Sensitivity of the number of doublets recovered on parameters of the algorithm for the female (*C*) and male (*D*) cell subsets. All other parameters were fixed at their default values while the parameter shown in each panel was varied. Boxplots summarize 10 repetitions at each parameter value.


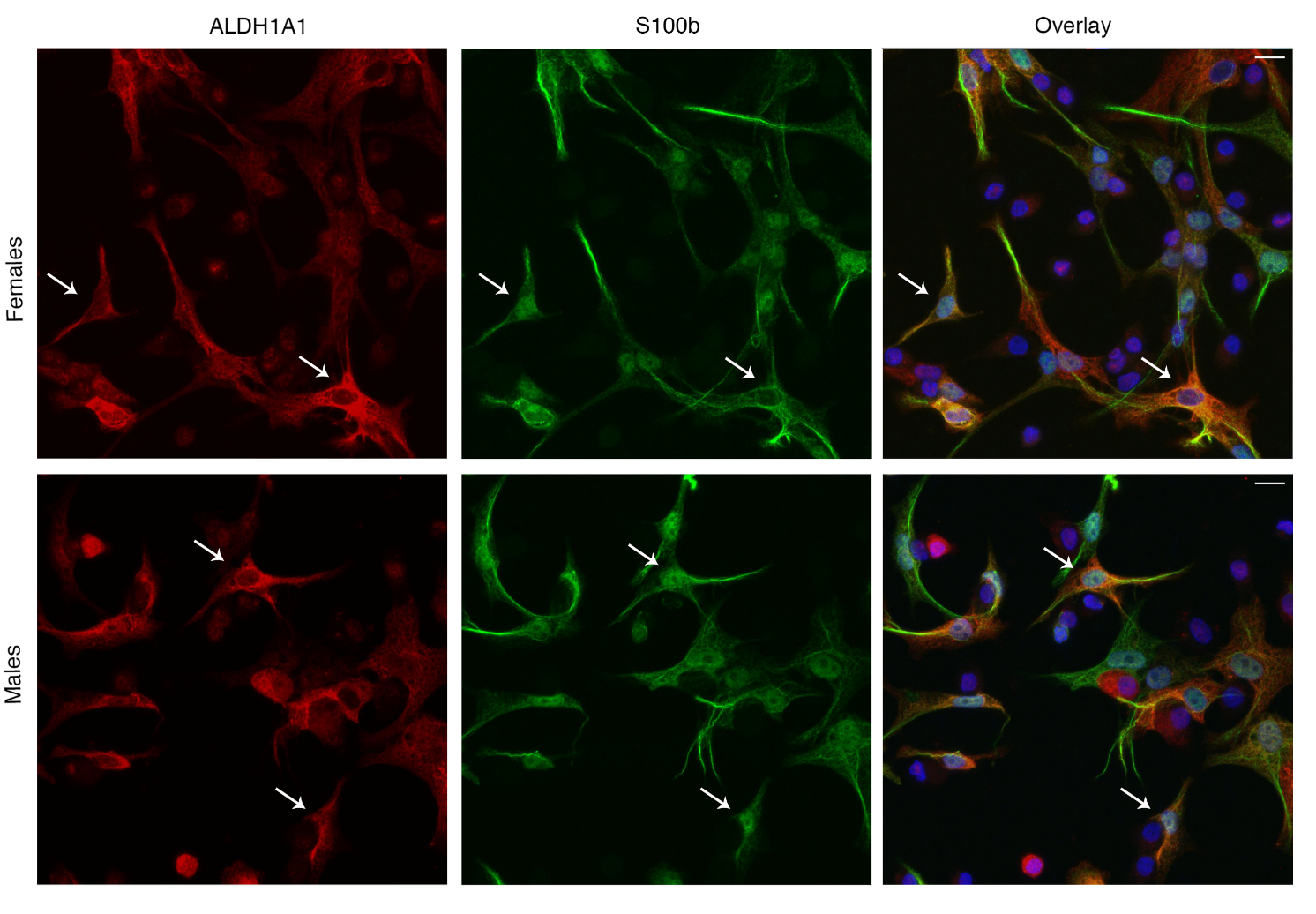


**Figure S3. Immunofluorescence analysis of sexual dimorphism in** **ALDH1A1 expression in anterior pituitary cells**. Expression of ALDH1A1 (red, left), S100B (green, center), and their overlay (right) in females (top panels) and males (bottom panels). Cell nuclei are stained with DAPI (blue). Arrows indicate example cells that coexpress both ALDH1A1 and S100B. Scale bars (applies to all images), 10 µm.


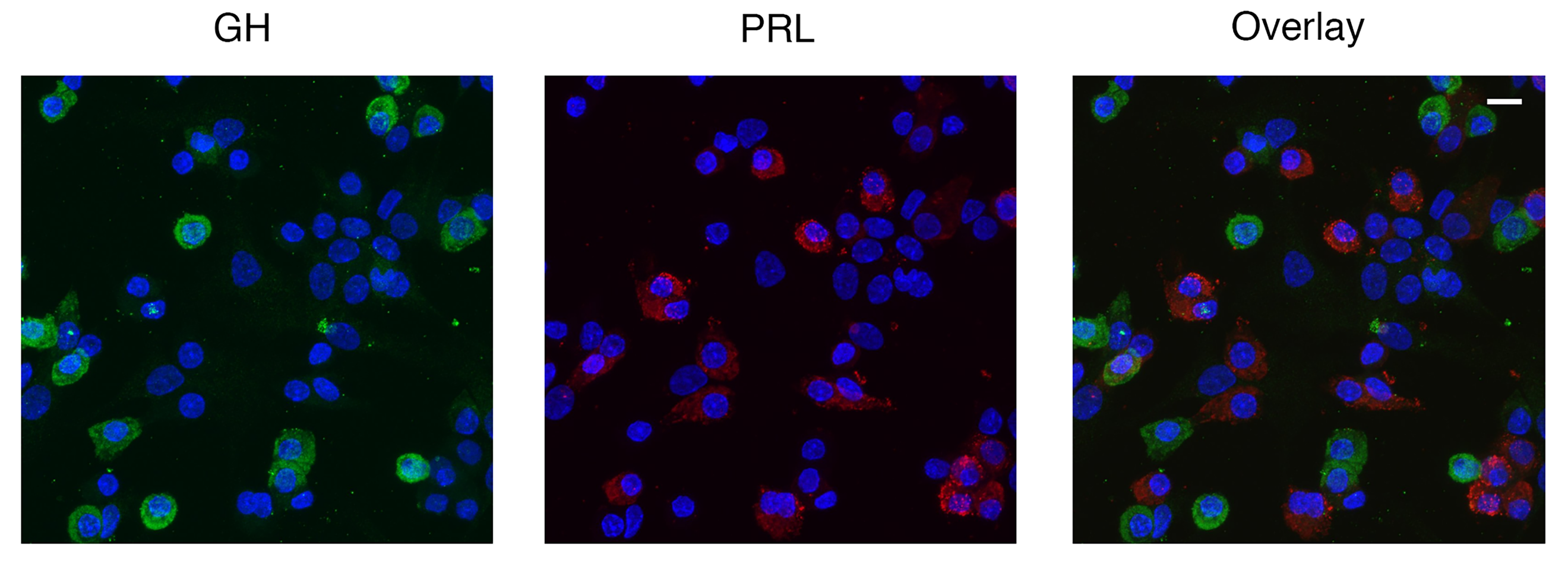


**Figure S4. Double-immunofluorescence labeling of GH and PRL in cultured anterior pituitary cells.** GH (*green, left*), PRL (*red, center*), and their overlay (*right*). Cell nuclei are labeled with DAPI (blue). Scale bar (applies to all images), 10 µm.

## Supplemental Tables

**Table S1. Percentage of each cell type expressing pituitary-dominant neuroendocrine marker genes.** For this and Table S2, genes are grouped into five categories based on their cell type-dominant or -specific expression. From top to bottom: FSC-specific, FSC-dominant, dominantly expressed in both FSC and HPC relative to non-pituitary cells, HPC-dominant (in at least one HPC type), and HPC-specific. Thr is the log_10_ expression threshold for each gene. Red colored values indicate genes expressed in at least 20% of cells of a given type, orange values indicate 5-20%.

|  | Gene | E | Le | EC | FSC | C | G | T | S | L | Thr |
| --- | --- | --- | --- | --- | --- | --- | --- | --- | --- | --- | --- |
| FSC-specific | *Nrgn* | 0.0 | 0.5 | 2.2 | 24.8 | 0.3 | 0.0 | 0.0 | 0.4 | 3.9 | 0.2 |
|  | *Olfml1* | 0.3 | 0.5 | 0.7 | 32.5 | 0.1 | 1.2 | 0.0 | 0.2 | 0.2 | 0.2 |
|  | *Tagln* | 0.0 | 0.2 | 0.7 | 45.9 | 0.1 | 0.0 | 0.0 | 0.1 | 0.0 | 0.5 |
| FSC-dominant | *Fgf13* | 0.0 | 0.4 | 0.7 | 26.1 | 5.8 | 9.8 | 5.6 | 0.4 | 2.9 | 0.2 |
|  | *Gpm6b* | 0.3 | 2.5 | 1.5 | 65.1 | 1.4 | 1.6 | 29.2 | 8.8 | 6.7 | 0.3 |
|  | *Metrn* | 0.3 | 1.4 | 4.4 | 39.8 | 1.1 | 0.8 | 7.9 | 1.1 | 1.2 | 0.2 |
|  | *Mxra8* | 0.0 | 0.5 | 21.2 | 55.7 | 0.6 | 0.4 | 1.1 | 0.4 | 0.0 | 0.2 |
|  | *Ndrg2* | 0.0 | 0.5 | 15.3 | 82.6 | 0.6 | 1.2 | 1.1 | 0.3 | 1.0 | 0.4 |
|  | *Pmp22* | 0.3 | 9.4 | 13.1 | 43.8 | 2.8 | 1.2 | 0.0 | 0.6 | 1.0 | 0.2 |
| FSC and HPC | *Apbb1* | 0.3 | 0.7 | 15.3 | 25.0 | 54.0 | 31.4 | 42.7 | 37.0 | 33.1 | 0.2 |
| coexpression | *Atxn10* | 0.5 | 18.9 | 42.3 | 55.2 | 83.1 | 51.8 | 44.9 | 49.4 | 48.2 | 0.2 |
|  | *Fuz* | 0.3 | 2.3 | 2.9 | 25.1 | 35.5 | 22.0 | 34.8 | 20.7 | 20.6 | 0.2 |
|  | *Gnas* | 0.0 | 11.9 | 80.3 | 91.6 | 96.4 | 86.5 | 94.4 | 96.0 | 98.2 | 1.2 |
|  | *Lrrn2* | 0.3 | 0.4 | 0.0 | 21.2 | 44.1 | 11.4 | 5.6 | 6.0 | 0.8 | 0.2 |
|  | *Mest* | 0.0 | 0.4 | 2.2 | 73.0 | 7.5 | 5.3 | 13.5 | 16.4 | 58.4 | 0.3 |
|  | *Nefl* | 0.0 | 0.9 | 1.5 | 39.8 | 0.1 | 68.6 | 24.7 | 1.6 | 1.6 | 0.3 |
|  | *Ngb* | 0.0 | 0.2 | 1.5 | 43.5 | 2.1 | 2.0 | 46.1 | 9.6 | 9.2 | 0.2 |
|  | *Nnat* | 0.0 | 0.2 | 0.7 | 59.6 | 52.9 | 31.8 | 37.1 | 5.5 | 6.5 | 0.6 |
|  | *Nptn* | 0.8 | 37.8 | 43.1 | 55.0 | 77.4 | 57.6 | 77.5 | 49.6 | 48.8 | 0.2 |
|  | *Nsg2* | 0.5 | 1.4 | 1.5 | 66.6 | 13.6 | 59.6 | 65.2 | 24.2 | 59.2 | 0.2 |
|  | *Ntrk2* | 0.8 | 0.5 | 1.5 | 71.4 | 61.1 | 6.9 | 16.9 | 12.8 | 2.4 | 0.3 |
|  | *Rtn1* | 0.0 | 3.6 | 6.6 | 68.8 | 31.4 | 26.1 | 47.2 | 52.7 | 71.0 | 0.3 |
|  | *Syt5* | 0.5 | 0.2 | 0.7 | 41.0 | 16.4 | 11.8 | 33.7 | 30.9 | 5.9 | 0.2 |
| HPC-dominant | *Aplp1* | 1.1 | 2.5 | 8.8 | 13.1 | 89.3 | 96.7 | 97.8 | 71.9 | 59.8 | 0.3 |
|  | *Bex2* | 0.0 | 0.7 | 0.0 | 19.9 | 99.1 | 95.9 | 94.4 | 99.4 | 93.3 | 0.6 |
|  | *Hap1* | 0.5 | 0.5 | 1.5 | 22.5 | 30.4 | 62.4 | 49.4 | 33.2 | 25.7 | 0.2 |
|  | *Lynx1* | 0.0 | 0.0 | 0.7 | 9.1 | 3.8 | 45.3 | 52.8 | 2.7 | 6.3 | 0.2 |
|  | *Manf* | 0.0 | 40.1 | 35.0 | 47.1 | 75.1 | 77.6 | 86.5 | 86.1 | 85.3 | 0.4 |
|  | *Ndn* | 0.5 | 1.1 | 4.4 | 37.4 | 73.8 | 60.0 | 59.6 | 44.2 | 22.7 | 0.2 |
|  | *Nell2* | 0.0 | 2.0 | 6.6 | 0.4 | 72.0 | 31.8 | 46.1 | 3.7 | 14.3 | 0.2 |
|  | *Ngfrap1* | 0.3 | 6.1 | 22.6 | 62.9 | 97.1 | 95.9 | 88.8 | 89.0 | 78.2 | 0.4 |
|  | *Olfm1* | 0.5 | 2.0 | 0.7 | 15.4 | 10.3 | 15.1 | 64.0 | 34.4 | 92.4 | 0.3 |
|  | *Pnma8b* | 0.5 | 0.9 | 0.0 | 26.1 | 81.8 | 58.4 | 85.4 | 54.8 | 56.3 | 0.2 |
|  | *Scg3* | 0.3 | 1.8 | 1.5 | 15.3 | 62.1 | 79.6 | 97.8 | 57.7 | 95.1 | 0.3 |
|  | *Scg5* | 0.0 | 0.5 | 0.7 | 10.8 | 98.6 | 98.8 | 94.4 | 97.2 | 98.4 | 0.6 |
|  | *Snca* | 29.9 | 0.2 | 0.0 | 2.7 | 79.4 | 1.2 | 19.1 | 1.4 | 31.4 | 0.3 |
|  | *Stmn1* | 0.0 | 3.6 | 20.4 | 19.9 | 97.3 | 40.4 | 93.3 | 57.6 | 64.5 | 0.6 |
|  | *Stxbp1* | 4.4 | 11.9 | 19.7 | 5.8 | 78.6 | 38.0 | 38.2 | 32.9 | 22.2 | 0.2 |
|  | *Syt1* | 0.3 | 0.5 | 2.9 | 10.4 | 51.1 | 2.0 | 27.0 | 36.7 | 14.9 | 0.2 |
|  | *Syt17* | 0.5 | 0.7 | 0.0 | 6.9 | 21.8 | 25.3 | 28.1 | 49.0 | 58.8 | 0.2 |
|  | *Sytl4* | 0.0 | 1.4 | 1.5 | 57.9 | 89.5 | 39.2 | 23.6 | 62.1 | 2.7 | 0.3 |
|  | *Tmem158* | 0.3 | 0.9 | 2.2 | 9.3 | 91.6 | 5.3 | 14.6 | 2.4 | 13.3 | 0.4 |
|  | *Tmem59l* | 0.3 | 2.0 | 1.5 | 15.5 | 38.8 | 84.1 | 92.1 | 36.5 | 88.4 | 0.3 |
| HPC- specific | *Caly* | 0.0 | 0.2 | 0.7 | 0.9 | 97.1 | 68.6 | 87.6 | 66.3 | 53.7 | 0.5 |
|  | *Chga* | 0.0 | 0.2 | 0.0 | 0.8 | 10.6 | 99.2 | 89.9 | 70.0 | 47.1 | 0.5 |
|  | *Chgb* | 0.0 | 0.4 | 0.0 | 0.6 | 98.5 | 92.2 | 97.8 | 70.8 | 98.4 | 0.6 |
|  | *Doc2g* | 0.0 | 0.0 | 0.0 | 0.1 | 92.4 | 0.0 | 0.0 | 0.0 | 0.2 | 0.5 |
|  | *Ina* | 0.0 | 0.7 | 0.7 | 0.4 | 85.1 | 50.6 | 37.1 | 28.7 | 21.4 | 0.2 |
|  | *Lrrn3* | 0.0 | 0.2 | 0.0 | 0.3 | 20.1 | 33.1 | 30.3 | 8.2 | 2.9 | 0.1 |
|  | *Nptxr* | 0.5 | 0.5 | 1.5 | 4.8 | 6.1 | 12.7 | 32.6 | 27.9 | 19.4 | 0.1 |
|  | *Nrg1* | 0.0 | 0.2 | 0.0 | 2.1 | 30.4 | 28.2 | 41.6 | 23.4 | 12.0 | 0.1 |
|  | *Nrsn1* | 0.0 | 0.7 | 0.7 | 0.9 | 4.0 | 57.6 | 66.3 | 57.8 | 24.9 | 0.2 |
|  | *Ntrk3* | 0.0 | 0.0 | 0.0 | 1.4 | 1.6 | 20.4 | 0.0 | 0.1 | 0.0 | 0.2 |
|  | *Pcp4* | 0.3 | 0.5 | 2.2 | 5.0 | 80.4 | 3.3 | 56.2 | 91.4 | 26.3 | 0.4 |
|  | *Pcsk1* | 0.0 | 1.8 | 0.0 | 0.4 | 14.4 | 18.0 | 53.9 | 35.8 | 26.5 | 0.2 |
|  | *Pcsk2* | 0.0 | 0.4 | 0.0 | 0.3 | 0.1 | 2.0 | 44.9 | 4.3 | 4.7 | 0.3 |
|  | *Resp18* | 0.0 | 0.2 | 0.7 | 0.8 | 99.9 | 98.8 | 98.9 | 99.3 | 96.1 | 1.0 |
|  | *Scg2* | 0.0 | 0.2 | 0.0 | 0.7 | 75.3 | 99.6 | 96.6 | 64.6 | 95.9 | 0.6 |
|  | *Snap25* | 0.3 | 1.6 | 0.7 | 2.3 | 97.8 | 80.8 | 82.0 | 59.3 | 57.3 | 0.3 |
|  | *Snap91* | 0.0 | 0.7 | 0.0 | 1.9 | 23.6 | 43.7 | 46.1 | 31.0 | 23.1 | 0.1 |
|  | *Sncb* | 0.0 | 0.4 | 0.7 | 0.4 | 72.8 | 17.1 | 25.8 | 8.3 | 28.2 | 0.2 |
|  | *Stmn2* | 0.3 | 0.4 | 0.7 | 0.7 | 36.5 | 66.5 | 78.7 | 6.9 | 7.1 | 0.2 |
|  | *Stmn3* | 0.0 | 0.7 | 2.9 | 1.5 | 94.0 | 78.0 | 87.6 | 48.2 | 61.6 | 0.3 |
|  | *Syp* | 0.5 | 0.4 | 0.0 | 0.5 | 46.8 | 46.1 | 62.9 | 45.3 | 22.9 | 0.2 |
|  | *Syt13* | 0.5 | 0.2 | 4.4 | 3.4 | 61.6 | 19.2 | 4.5 | 28.8 | 15.3 | 0.2 |
|  | *Syt14* | 0.3 | 0.2 | 0.0 | 1.5 | 35.8 | 21.2 | 23.6 | 18.3 | 22.2 | 0.1 |
|  | *Syt4* | 0.0 | 1.3 | 1.5 | 3.9 | 76.3 | 66.5 | 76.4 | 53.9 | 31.4 | 0.2 |
|  | *Syt7* | 0.5 | 0.7 | 0.7 | 3.1 | 23.4 | 30.2 | 57.3 | 71.1 | 59.8 | 0.2 |
|  | *Tubb3* | 0.0 | 0.9 | 0.0 | 1.2 | 15.1 | 63.3 | 32.6 | 37.3 | 65.9 | 0.2 |
|  | *Uchl1* | 0.3 | 2.2 | 1.5 | 3.2 | 93.8 | 98.8 | 98.9 | 94.3 | 80.2 | 0.4 |

**Table S2.** **Percentage of each cell type expressing pituitary-dominant genes encoding endogenous ligands**. Genes are categorized and colored as in Table S1.

|  | Gene | E | Le | EC | FSC | C | G | T | S | L | Thr |
| --- | --- | --- | --- | --- | --- | --- | --- | --- | --- | --- | --- |
| FSC-specific | *Cxcl12* | 0.0 | 0.7 | 3.6 | 66.5 | 1.3 | 0.0 | 1.1 | 1.2 | 0.4 | 0.3 |
|  | *Edn3* | 0.0 | 2.7 | 0.7 | 48.9 | 0.0 | 0.4 | 1.1 | 0.2 | 0.2 | 0.3 |
|  | *Gpha2* | 0.0 | 0.0 | 0.7 | 36.7 | 1.3 | 0.4 | 0.0 | 0.1 | 0.2 | 0.8 |
|  | *Mdk* | 0.0 | 0.4 | 0.7 | 82.2 | 0.5 | 1.6 | 3.4 | 1.1 | 1.0 | 0.4 |
|  | *Penk* | 0.0 | 0.0 | 0.7 | 81.6 | 0.0 | 0.0 | 1.1 | 0.2 | 1.0 | 0.6 |
|  | *Rarres2* | 0.0 | 0.2 | 3.6 | 22.4 | 0.3 | 0.0 | 3.4 | 0.2 | 0.4 | 0.2 |
|  | *Wnt5a* | 0.0 | 0.0 | 1.5 | 32.7 | 0.9 | 2.4 | 0.0 | 0.2 | 0.0 | 0.2 |
| FSC-dominant | *Anxa1* | 0.0 | 6.3 | 20.4 | 76.3 | 0.0 | 0.0 | 2.2 | 0.3 | 5.1 | 0.3 |
|  | *Ctf1* | 0.0 | 0.5 | 1.5 | 32.4 | 1.1 | 2.0 | 6.7 | 0.2 | 1.6 | 0.2 |
|  | *Efnb3* | 0.0 | 0.5 | 0.7 | 46.2 | 0.8 | 2.4 | 5.6 | 0.3 | 0.6 | 0.3 |
|  | *Fgf13* | 0.0 | 0.4 | 0.7 | 26.1 | 5.8 | 9.8 | 5.6 | 0.4 | 2.9 | 0.2 |
|  | *Igfbp2* | 0.0 | 0.2 | 5.8 | 31.2 | 0.4 | 5.7 | 2.2 | 0.3 | 0.0 | 0.4 |
|  | *Il33* | 0.0 | 3.4 | 5.1 | 70.4 | 0.1 | 0.0 | 0.0 | 0.2 | 0.0 | 0.3 |
|  | *Ptn* | 0.0 | 0.0 | 8.8 | 88.3 | 1.3 | 0.4 | 2.2 | 0.5 | 6.9 | 0.5 |
|  | *Tgfb2* | 0.0 | 0.2 | 5.8 | 33.3 | 1.3 | 0.4 | 3.4 | 0.4 | 0.0 | 0.2 |
| FSC and HPC | *Angpt1* | 0.0 | 0.5 | 0.0 | 29.5 | 18.6 | 1.2 | 4.5 | 17.3 | 10.4 | 0.2 |
| coexpression | *Anxa5* | 0.0 | 8.8 | 43.1 | 86.4 | 54.6 | 58.4 | 22.5 | 35.2 | 13.3 | 0.5 |
|  | *Anxa7* | 1.4 | 27.3 | 28.5 | 39.5 | 68.1 | 40.8 | 41.6 | 28.8 | 30.4 | 0.2 |
|  | *Ccl27* | 0.0 | 0.9 | 1.5 | 21.8 | 38.9 | 32.2 | 36.0 | 33.9 | 33.9 | 0.2 |
|  | *Cntn1* | 0.5 | 0.9 | 1.5 | 37.0 | 55.8 | 2.9 | 47.2 | 36.4 | 10.0 | 0.2 |
|  | *Copa* | 0.0 | 16.2 | 21.9 | 26.4 | 51.5 | 46.1 | 56.2 | 44.5 | 32.9 | 0.2 |
|  | *Efna5* | 0.0 | 0.2 | 8.0 | 24.3 | 40.1 | 29.4 | 27.0 | 16.5 | 9.2 | 0.2 |
|  | *Lgals1* | 0.0 | 11.9 | 8.8 | 48.6 | 2.4 | 44.1 | 30.3 | 4.2 | 57.1 | 0.3 |
|  | *Lrrc4b* | 0.0 | 0.2 | 0.0 | 13.1 | 29.4 | 5.3 | 4.5 | 5.7 | 7.8 | 0.2 |
|  | *Tgfb3* | 0.0 | 0.5 | 2.2 | 12.8 | 27.0 | 1.2 | 1.1 | 5.8 | 7.6 | 0.2 |
|  | *Vegfb* | 0.0 | 5.4 | 5.1 | 20.1 | 18.6 | 26.9 | 25.8 | 20.8 | 11.2 | 0.2 |
| HPC-dominant | *C1qtnf4* | 0.5 | 0.4 | 0.7 | 6.2 | 63.1 | 53.9 | 34.8 | 9.3 | 27.1 | 0.2 |
|  | *Cxcl14* | 0.5 | 2.2 | 1.5 | 7.5 | 81.9 | 4.5 | 10.1 | 70.3 | 66.7 | 0.3 |
|  | *Fgf9* | 0.0 | 0.5 | 0.0 | 5.4 | 24.8 | 24.9 | 34.8 | 40.8 | 22.7 | 0.2 |
|  | *Inha* | 0.3 | 0.9 | 0.0 | 6.3 | 1.4 | 26.5 | 10.1 | 9.2 | 9.2 | 0.1 |
|  | *Spp1* | 0.0 | 7.2 | 0.0 | 0.6 | 0.3 | 27.3 | 1.1 | 0.2 | 0.4 | 0.4 |
|  | *Vegfa* | 0.0 | 3.2 | 0.7 | 12.5 | 1.3 | 45.3 | 11.2 | 2.6 | 1.8 | 0.2 |
| HPC- specific | *Artn* | 0.3 | 0.2 | 0.7 | 1.2 | 12.9 | 3.3 | 30.3 | 24.8 | 16.9 | 0.1 |
|  | *Bmp15* | 0.0 | 0.0 | 0.7 | 3.7 | 0.3 | 10.2 | 47.2 | 0.3 | 8.4 | 0.2 |
|  | *Dlk1* | 0.0 | 0.2 | 0.0 | 0.3 | 6.0 | 1.2 | 71.9 | 99.6 | 20.0 | 0.9 |
|  | *Fgf14* | 0.0 | 0.0 | 0.0 | 0.5 | 20.3 | 3.7 | 9.0 | 2.6 | 0.8 | 0.1 |
|  | *Gal* | 0.0 | 0.2 | 2.9 | 1.4 | 0.1 | 0.4 | 4.5 | 7.3 | 27.3 | 0.4 |
|  | *Icam5* | 0.0 | 3.6 | 2.2 | 0.3 | 1.5 | 22.4 | 1.1 | 2.5 | 0.8 | 0.2 |
|  | *Il22* | 0.0 | 0.0 | 0.0 | 0.0 | 28.3 | 0.4 | 0.0 | 0.4 | 1.8 | 0.2 |
|  | *Nmb* | 0.3 | 0.0 | 0.0 | 1.1 | 20.0 | 14.3 | 19.1 | 6.4 | 8.2 | 0.2 |
|  | *Nmu* | 0.0 | 1.1 | 0.0 | 0.9 | 9.3 | 21.2 | 70.8 | 12.3 | 2.2 | 0.3 |
|  | *Nog* | 0.0 | 0.2 | 0.0 | 0.8 | 0.1 | 1.6 | 0.0 | 30.5 | 5.5 | 0.1 |
|  | *Pdyn* | 0.0 | 0.0 | 0.0 | 3.1 | 5.1 | 31.0 | 0.0 | 2.1 | 1.8 | 0.2 |
|  | *Wnt10a* | 0.0 | 0.2 | 0.0 | 0.2 | 3.0 | 0.4 | 1.1 | 20.2 | 10.0 | 0.2 |

**Table S3 - Mitotic cell cycle genes expressed in pituitary cells**. A set of 39 mitotic cell cycle marker genes could be identified using an iterative process, beginning with a known curated set of marker genes (Materials and Methods). Cells expressing at least 14 of these genes were considered as proliferating (108 cells). Cell cycle marker genes satisfied the criteria: significantly upregulated (adjusted-P < 0.001) genes relative to non-proliferating cells, at least 50% proliferating cells expressing > threshold, and less than 5% expressing in the remaining cells, and were annotated with the Gene Ontology term “mitotic cell cycle” (GO:0000278).

*Aurka, Aurkb, Birc5, Ccna2, Ccnb1, Cdc20, Cdca8, Cdk1, Cdkn3, Cenpf, Cenpt, Cenpw, Ckap2, Cks2, Ect2, Fbxo5, Kif11, Kif20a, Kif20b, Kif23, Kifc1, Knstrn, Mad2l1, Mki67, Ncapg, Ndc80, Nuf2, Nusap1, Pbk, Plk1, Pttg1, Sapcd2, Ska1, Spc25, Tacc3, Top2a, Tpx2, Ttk, Ube2c.*

**Table S4. Comparison of qRT-PCR analysis of expression of selected genes in anterior pituitary tissues and single anterior pituitary cells with scRNAseq done in single anterior pituitary cells.** Data shown for qRT-PCR are normalized values (mean ± SEM; six diestrus female pituitaries and six samples from dispersed anterior pituitary cells from random cycling females) using *Gapdh* as a reference gene. Data for scRNAseq show the mean ± SEM of expression (normalized counts) for all female cells. Both qRT-PCR and scRNA seq analyses were performed with cells 30 min after dispersion.

| Gene | qRT-PCR, Pituitary Tissue | | | qRT-PCR, Dispersed cells | | | scRNAseq | | |
| --- | --- | --- | --- | --- | --- | --- | --- | --- | --- |
| *Caly* | 26.26 | ± | 3.22 | 16.98 | ± | 0.29 | 2.68 | ± | 0.08 |
| *Cga* | 347.20 | ± | 22.98 | 321.81 | ± | 9.77 | 44.10 | ± | 2.76 |
| *Fshb* | 4.54 | ± | 1.23 | 5.64 | ± | 0.10 | 0.55 | ± | 0.17 |
| *Gata2* | 3.18 | ± | 0.39 | 2.50 | ± | 0.05 | 0.17 | ± | 0.02 |
| *Gfap* | 0.12 | ± | 0.07 | 0.04 | ± | 0.01 | 0.001 | ± | 0.001 |
| *Gh1* | 15543.02 | ± | 1140.43 | 13625.84 | ± | 225.57 | 621.77 | ± | 20.27 |
| *Lhb* | 195.32 | ± | 14.31 | 239.47 | ± | 4.63 | 24.32 | ± | 1.66 |
| *Lhx3* | 1.56 | ± | 0.12 | 1.32 | ± | 0.04 | 0.29 | ± | 0.01 |
| *Lhx4* | 0.02 | ± | 0.004 | 0.03 | ± | 0.002 | 0.03 | ± | 0.003 |
| *Pitx3* | 0.32 | ± | 0.06 | 0.23 | ± | 0.01 | 0.06 | ± | 0.01 |
| *Pomc* | 618.41 | ± | 32.03 | 547.16 | ± | 23.88 | 182.32 | ± | 10.02 |
| *Prl* | 21436.61 | ± | 2254.46 | 22803.86 | ± | 520.92 | 202.15 | ± | 9.96 |
| *S100b* | 3.78 | ± | 0.33 | 8.57 | ± | 0.22 | 4.12 | ± | 0.12 |
| *Sez6l2* | 30.46 | ± | 2.17 | 33.76 | ± | 0.74 | 0.57 | ± | 0.02 |
| *Snap25* | 15.16 | ± | 1.13 | 15.07 | ± | 0.52 | 1.27 | ± | 0.04 |
| *Sox2* | 0.28 | ± | 0.04 | 0.84 | ± | 0.02 | 0.65 | ± | 0.02 |
| *Stmn3* | 4.92 | ± | 0.40 | 3.00 | ± | 0.08 | 1.01 | ± | 0.03 |
| *Tmem130* | 44.95 | ± | 3.01 | 40.52 | ± | 0.77 | 1.48 | ± | 0.04 |
| *Tshb* | 76.25 | ± | 9.89 | 31.74 | ± | 0.79 | 2.22 | ± | 1.12 |
